# Ultraviolet-water-induced angstrom-sized channels in membrane for precise ion sieving

**DOI:** 10.1093/nsr/nwaf404

**Published:** 2025-09-22

**Authors:** Yaxiong Cheng, Baochun Meng, Huijun Yao, Haijian Shi, Guozhen Liu, Guining Chen, Gongping Liu, Wanqin Jin, Nanping Xu

**Affiliations:** State Key Laboratory of Materials-Oriented Chemical Engineering, College of Chemical Engineering, Nanjing Tech University, Nanjing 211816, China; State Key Laboratory of Materials-Oriented Chemical Engineering, College of Chemical Engineering, Nanjing Tech University, Nanjing 211816, China; Institute of Modern Physics, Chinese Academy of Sciences, Lanzhou 730000, China; State Key Laboratory of Materials-Oriented Chemical Engineering, College of Chemical Engineering, Nanjing Tech University, Nanjing 211816, China; State Key Laboratory of Materials-Oriented Chemical Engineering, College of Chemical Engineering, Nanjing Tech University, Nanjing 211816, China; State Key Laboratory of Materials-Oriented Chemical Engineering, College of Chemical Engineering, Nanjing Tech University, Nanjing 211816, China; State Key Laboratory of Materials-Oriented Chemical Engineering, College of Chemical Engineering, Nanjing Tech University, Nanjing 211816, China; Suzhou Laboratory, Suzhou 215125, China; State Key Laboratory of Materials-Oriented Chemical Engineering, College of Chemical Engineering, Nanjing Tech University, Nanjing 211816, China; Suzhou Laboratory, Suzhou 215125, China

**Keywords:** polymer membrane, angstrom-sized channel, hydroxyl radical, radical reaction, ion separation

## Abstract

Membranes with angstrom-sized channels can enable precise ion separation. While progress has been achieved in polymer membranes, which are the dominant commercial membranes, significant technical challenges of creating angstrom-sized channels remain in these membranes. Here, we report an ultraviolet-water (UV-W) strategy to create angstrom-sized channels (2.9–7.8 Å) with tunable size and charges in ion-tracked polymer membranes, where ‘UV-W’ refers to water illuminated by ultraviolet light, which is able to generate highly oxidative hydroxyl radicals. These generated hydroxyl radicals can scissor polymer chains within the ion tracks. Thus, we first create water passages within the polymer, then with the aid of hydroxyl radicals we subsequently form transport channels. The resultant membrane exhibits outstanding separation performance with a Li^+^ permeation rate of 0.3 mol m^−2^ h^−1^ and Li^+^/Mg^2+^ selectivity of 1024, which surpassed the performance of the existing polymer membranes. This easy approach provides an avenue for developing angstrom-sized channels in diverse polymers, offering broad application potential in clean energy fields.

## Teaser

This study utilizes the photochemical property of water molecules to generate hydroxyl radicals under UV light, which can scissor polymer chains to create angstrom-sized channels in polymer membranes, enabling precise ion sieving.

## INTRODUCTION

Membranes capable of precisely separating ions and small molecules offer great prospects for industrial applications, including clean energy production and water desalination [1–8]. Membrane-based separation techniques have been applied to extract mineral resources such as U and Li from saltwater resources [9,10], a process with broad applications in the fields of nuclear fission and rechargeable Li-ion batteries [11–13]. To achieve highly efficient separation, the membrane channels must be formed with angstrom-scale precision [14,15]. While emerging materials like graphene, metal–organic frameworks (MOFs) and covalent organic frameworks (COFs) demonstrate exceptional sub-nanometer channel precision [16–25], their scalability remains challenging for industrial preparation. Polymer membranes (>70% share of the separation membrane market) [26] are widely used for separation tasks but typically face a trade-off between permeability and selectivity [27–29]. This is due to the non-uniform and dynamic behavior of the free volume cavities formed by the packing of polymer chains.

Attempts have explored the incorporation of sub-nanochannels into polymer membranes to improve the separation performance [30,31]. Phase inversion is the most widely used technique to prepare polymer membranes, resulting in a variety of morphologies suitable for different applications. Such morphologies range from microporous filtration membranes to denser nanofiltration and reverse osmosis membranes [32]. However, the pore size distribution of the barrier layer in polymer membranes prepared by phase transition is usually broad and lacks uniform sub-nanochannels [33,34], thus limiting size-based separation selectivity. Ion irradiation could also generate a track, i.e. a narrow damage trail in polymers, and it cannot form sub-nanochannels directly (Fig. S1 in the online Supplementary data) [35,36]. The tracks usually require post-processing to form interconnected channels. Traditional post-processing methods mainly involve chemical etching or UV treatment to sensitize the latent tracks for quick chemical etching. During the chemical etching process, the pore geometry can be controlled, with pore density independent of pore size [37]. The scale of channel formed by chemical etching on the ion-irradiated membranes is normally in the nanometer range. Wang’s group reported the landmark ‘track-UV’ strategy for constructing angstrom-scale channels in ion-irradiated membranes [38], enabling precise molecular separation [35,39,40]. This approach relies on heavy projectile ions (e.g. Ta, Bi, Au) and direct photo-oxidation of the polymer fragments within the track in an air environment to generate the channels. Leveraging this concept, we sought a milder, more controllable etching medium for angstrom-scale channel formation in polymer membranes.

**Figure 1.**
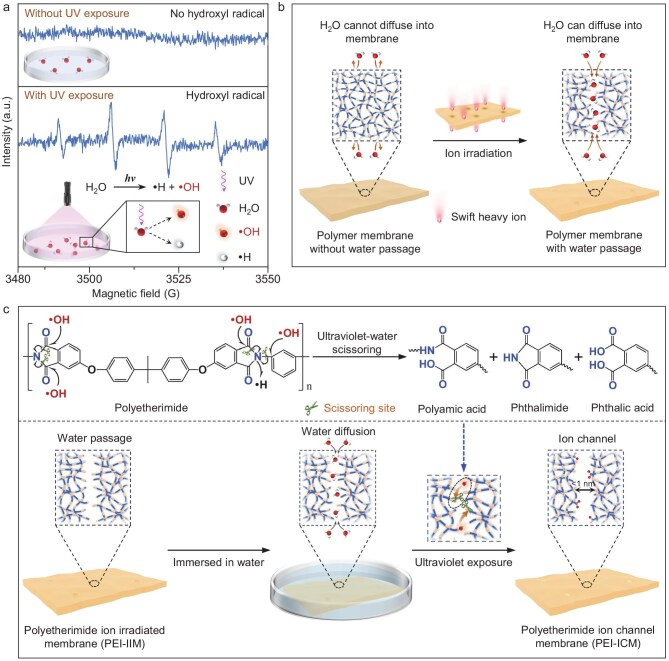
Schematic of UV-W process. (a) EPR spectra of (top) water without UV exposure and (bottom) water under UV exposure. (b) Route of water molecules diffusing into the interior of the polymer membranes. (c) Schematic of the PEI-ICM prepared by the UV-W process.

Water molecules illuminated by UV could serve as a controllable medium for creating channels in polymer membranes. When water molecules absorb UV light, they undergo homolysis and photochemical ionization, leading to the production of reactive oxygen species (ROS), particularly oxygen-containing free radicals with unpaired electrons, which are extremely chemically reactive [41,42]. The highly oxidizing hydroxyl radicals (·OH) are a major type of reactive oxygen species produced under UV irradiation, which can scissor polymer chains [43–45]. We confirmed the generation of hydroxyl radicals by electron paramagnetic resonance (EPR) spectroscopy (Fig. 1a), which showed that no characteristic hydroxyl radical signal peak appeared when the water was not exposed to UV light. In contrast, the characteristic hydroxyl radical signal peak appeared when water was exposed to UV. We call the water illuminated by UV light ‘UV-water’ (UV-W). Simultaneously, we envisaged that, by introducing UV-W into the interior of the polymer such that water molecules diffuse into this interior, angstrom-sized channels eventually could be formed with the help of hydroxyl radicals within the membrane. The passage of water molecules into the membrane can be achieved through ion irradiation or swelling of the membrane (Fig. 1b, Fig. S2). Inspired by these mechanisms, we therefore proposed an *in situ* UV-W scissoring approach to form angstrom-sized channels in a polymer membrane for ion separation, as shown in Fig. 1c.

To demonstrate the strategy, we chose a polyetherimide ion-irradiated membrane (PEI-IIM) as a precursor, which was obtained by subjecting a polyetherimide membrane (PEIM) to ion irradiation. When the membrane is immersed in water, the water molecules diffuse into the interior of the PEI-IIM. Upon UV light exposure, the water molecules photolyze to produce hydroxyl radicals, which actively scissor the surrounding polymer chains and lead to scission of chains and the formation of new functional groups [46], ultimately forming angstrom-sized channels inside the PEI ion channel membrane (PEI-ICM). By controlling the UV exposure time, the channel sizes can be tuned at the angstrom scale. The adjustable sizes and functional groups of the angstrom-sized channels formed in the polymer membrane are capable of sieving monovalent ions (e.g. Li^+^, K^+^, Na^+^) from multivalent ions (e.g. Mg^2+^, Ca^2+^, La^3+^).

## RESULTS AND DISCUSSION

### Preparation of membranes by the UV-W process

**Figure 2.**
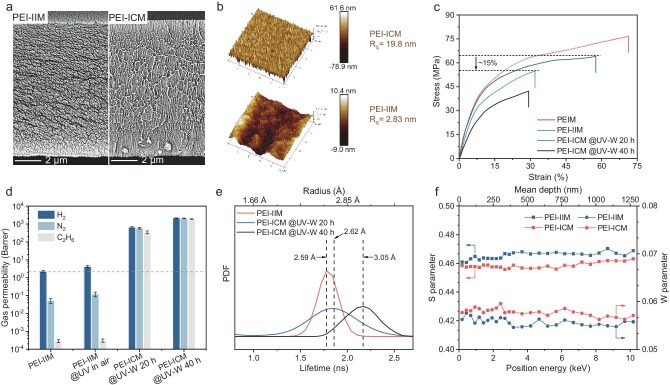
Morphologies and channel properties of membranes. (a) Cross-sectional SEM images of the (left) PEI-IIM and (right) PEI-ICM. (b) AFM images of the (bottom) PEI-IIM and (top) PEI-ICM. The surface roughness of PEI-IIM and PEI-ICM is 2.83 and 19.8 nm, respectively. (c) Stress–strain curves of the PEIM, PEI-IIM and PEI-ICM. (d) Gas permeabilities of membranes prepared under different conditions at 30°C and 1 bar. (e) PALS of PEI-IIM and PEI-ICM with the UV-W process for 20 and 40 h. (f) Slow-positron DBES of PEI-IIM and PEI-ICM.

The precursor of the PEI-ICM is PEI-IIM, which is prepared by irradiating 10-μm thick PEI membranes (Figs S2 and S3, Table S1). The energetic ion bombardment leads to changes in the properties of the membrane, including its colour from clear amber to yellow (Fig. S4a and b), a break in the transverse structure of the cross-section (Fig. S4d and e) and a slight increase in the roughness of the membrane surface (Fig. S4g and h). After the UV-W process, the cross-section exhibits a vertically oriented crack structure, which is in marked contrast to the initial PEI-IIM (Fig. 2a, Fig. S4f), and the surface roughness of the membrane significantly increases (Fig. 2b, Fig. S4c and i). We used dynamic mechanical analysis to test the stress–strain curves of the membranes; the result shows that the tensile strength and elongation at the break of the PEI-ICM decreased (Fig. 2c). However, the membrane retains sufficient tensile strength and flexibility to support its use in ion separation applications. The evolution of the morphological and mechanical properties of the membrane reveals that the UV-W process induces *in situ* scissoring, not only on the surface to increase roughness but also within the membrane to generate ion channels (PEI-ICM). We used an array of gas molecules with varying kinetic diameters to probe the channel formation and size at the angstrom scale [2,47,48]. As shown in Fig. 2d, both PEI-IIM and PEI-IIM treated by UV exposure in air showed extremely low gas permeability, while the gas permeability of PEI-ICM with the UV-W process for 20 h increased rapidly. The trend suggests that the UV-W process successfully created the channels with adjustable sizes. Meanwhile, we used positron annihilation lifetime spectroscopy (PALS) and slow-positron Doppler broadening energy spectroscopy (DBES) to determine the variation of channel size in the PEI-IIM and PEI-ICM treated by different UV-W times. The right shift of the positron lifetime indicated an increase in membrane channel sizes during the UV-W process (Fig. 2e). DBES shows that the reduced *S*-parameter and enhanced *W*-parameter in PEI-ICM are relative to PEI-IIM (Fig. 2f). This contrast arises from the UV-W process eliminating isolated free-volume cavities to form continuous oxygen-functionalized channels within the ion-irradiated portion of the membrane. Concurrent UV-induced cross-linking in the non-irradiated portion further changes the annihilation microenvironment, thereby suppressing low-momentum positron annihilation events. Moreover, we washed the PEI-ICM with deionized water after soaking it in KCl solution, and then we sliced the PEI-ICM to perform energy-dispersive X-ray spectroscopy (EDS) mapping. The results showed that K ions were present in the internal channels of the PEI-ICM (Fig. S5), which further indicates that the UV-W process can form channels in the interior of membranes.

**Figure 3.**
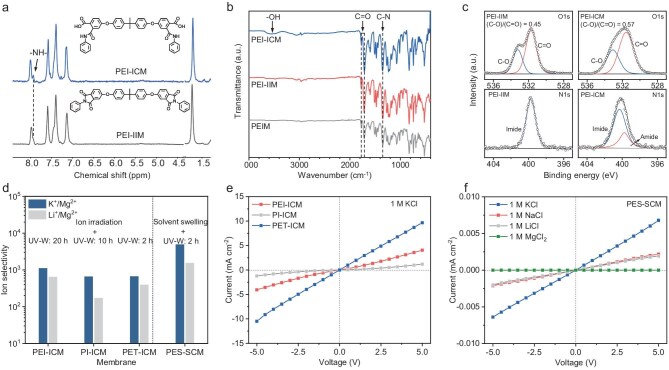
Chemical properties and universality of UV-W process. (a) ^1^H NMR spectra of the (bottom) PEI-IIM and (top) PEI-ICM. (b) FTIR-ATR spectra of PEIM, PEI-IIM and PEI-ICM. (c) XPS spectra of PEI-IIM and PEI-ICM. (d) K^+^/Mg^2+^ and Li^+^/Mg^2+^ selectivity performance of membranes fabricated through the ion irradiation @UV-W process and solvent swelling @UV-W process. *I−V* curves of membranes fabricated by (e) the ion irradiation @UV-W process and (f) the solvent swelling @UV-W process in 1 M salt solution.

The UV-W process also alters the chemical structure of the membranes, encompassing the chemical composition, surface functional groups and hydrophilicity, to produce the ion transport channels. Under the UV light, polyamic acid, polyphthalimide and polyphthalic acid were produced respectively via free radical reactions in the presence of hydroxyl radicals generated from water by attacking the C=O and C–N bonds in the PEI molecule (Fig. 1a and c). In the ^1^H nuclear magnetic resonance (^1^H NMR) spectrum of PEI-ICM, a singlet for −NH− of N–H phthalimide groups and polyamic acid resonated at a chemical shift of 7.93 ppm (Fig. 3a, Fig. S6). Compared with PEI-IIM and PEI-ICM, a red shift from 1720 to 1712 cm^−1^ of C=O groups were found in the Fourier transform infrared attenuated total reflectance (FTIR-ATR) spectra (Fig. S7), confirming that the phthalimide units undergo bond cleavage, leading to a structure of polyamic acids, a longer conjugated chain connected to the C=O group. Additionally, the FTIR-ATR spectra vibrated at 3490 cm^−1^ (Fig. 3b), indicating the carboxylic acid −OH group of PEI-ICM. X-ray photoelectron spectrometry (XPS) and thermogravimetric-mass spectrometry (TG-MS) results also confirm the products produced by the hydroxyl radical reaction. O1s XPS shows that the C–O/C=O ratio increases from 0.45 to 0.57, revealing that the C–O content increased in the PEI-ICM membrane treated by the UV-W process, and further indicating the elevation in the content of carboxyl groups (Fig. 3c). At a binding energy (BE) of 399.8 eV, N1s XPS shows characteristic signals of C–N in amide groups in the PEI-ICM membrane, which demonstrates the generation of polyamic acid. Meanwhile, the formation of phthalic acid, polyamic acid and N–H phthalimide during the UV-W process could generate CO_2_ and NH_3_ as gaseous by-products (Fig. S8), as evidenced by the ion current corresponding to HBr from TG-MS. Moreover, with the generation of -COOH, the hydrophilicity of the membrane increases, which was confirmed by the decrease in contact angle from 90° to ∼65° (Fig. S9). This result is consistent with the surface roughness (Fig. 2b).

On the foundation of the PEI-ICM preparation mechanism, as shown in Fig. 1b and Fig. S1, water molecules diffuse into the interior of a membrane that is treated by either ion irradiation or solvent welling. This allows us to use different polymer membranes to form sub-nanochannels via the UV-W process. Similar to the fabrication of the PEI-ICM through the ion irradiation @UV-W process, we exposed the ion-irradiated polyimide (PI) and polyethylene terephthalate (PET) membranes immersed in water to the UV-W process, resulting in the formation of the PI-ICM and PET-ICM, respectively (Fig. S10a). Notably, in polymers such as PI and PET, which are highly susceptible to UV-induced photolysis, channel formation may also proceed via a track-UV mechanism. In this process, hydroxyl radicals and UV irradiation act in concert to cleave polymer chains within the track region of the ion-irradiated membrane. Meanwhile, we used a dimethyl sulfoxide/water mixed solution (DMSO:H_2_O = 5:2) to swell a 25-µm thick commercial polyethersulfone (PES) membrane to introduce free volumes for water molecules diffusing into the polymer membrane. Subsequently, the swollen PES membrane was immersed in water for the UV-W process to prepare the PES swelling channel membrane (PES-SCM) (Figs S10b and S11).

The UV-W process within membranes was effectively controlled by modulating the respective UV exposure duration to introduce highly charged angstrom-sized channels in membranes. We analyzed the transport properties of ions in these membranes (Figs S12 and S13). The ion transport properties were reflected by current−voltage (*I*−*V*) curves (Fig. S14), which were measured in salt solutions. As shown in Fig. 3d–f, the K^+^/Mg^2+^ and Li^+^/Mg^2+^ selectivity of the PEI-ICM, PI-ICM and PET-ICM were lower than that of the PES-SCM, while the KCl ion current of the ICM was three orders of magnitude higher than that of the PES-SCM. The result shows that the ion irradiation technology allows the polymer membranes to contain straight-through water passages, enabling the membranes fabricated by ion irradiation @UV-W to have shorter ion channels compared to the membranes fabricated by solvent swelling @UV-W. Integrating the ion permeability and selectivity of the membranes, we systematically investigated the effect of UV-W on the properties of the PEI-ICM.

**Figure 4.**
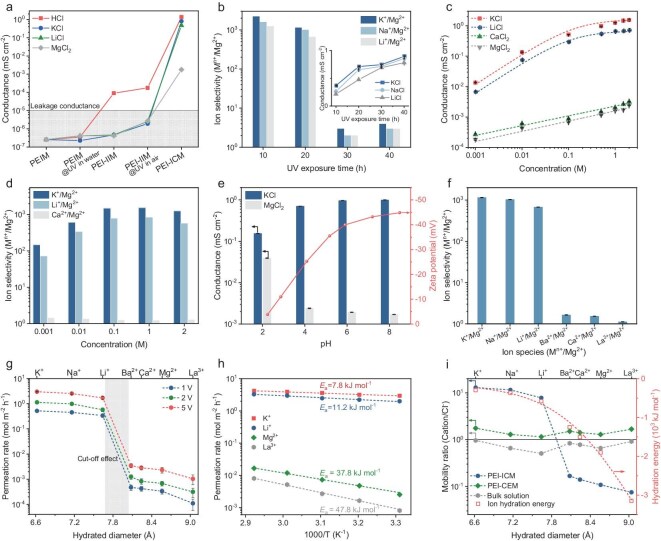
Single-ion transport property of PEI-ICM. (a) Ion conductance of membranes prepared under different conditions measured in 1 M salt solutions. (b) Ion selectivity of PEI-ICM under different UV illumination times. The inset is ion conductance as a function of UV illumination times. (c) Ion conductance of PEI-ICM as a function of salt concentration. (d) Ion selectivity of PEI-ICM at different salt concentrations. PEI-ICM exhibits the highest ion selectivity at 1 M. (e) Ion conductance and zeta potential of PEI-ICM as a function of solution pH. (f) Ion selectivity of PEI-ICM at 1 V. (g) Permeation rates of various ions through PEI-ICM as a function of the ion hydrated diameter. (h) Temperature dependence of ion permeation in PEI-ICM. (i) Cation mobility ratios calculated from drift-diffusion measurements.

To study the channel formation of the PEI-ICM, we analyzed the transport properties of ions and protons through the membranes prepared by different conditions (Fig. 4a, Fig. S15). The ion and proton transport properties are reflected by the ion conductance (G). As shown in Fig. 4a, all ionic conductivities of the pristine PEI membranes and PEI membranes treated with the UV-W process for 20 h exhibited extremely low values of G (<10^−5^ mS cm^−2^), suggesting the absence of water passages and ion channels within both membranes. After Kr ion irradiation, the conductance of protons in the PEI-IIM showed a notable increase (∼10^−4^ mS cm^−2^), while the conductivity of metal ions remained at extremely low values, demonstrating that only continuous water passages are formed within the PEI-IIM. The proton and metal ion conductance in the membrane only slightly increased after approximately 20 h of UV exposure in air, indicating that simple UV processing was unable to form channels in the PEI-IIM. Following a UV-W process of 20 h, the conductance of both protons and metal ions in the membrane increased significantly, especially for monovalent metal ions, which showed an increase of approximately six orders of magnitude compared to PEI-IIM. Meanwhile, the conductance of divalent ions only increased by about three orders of magnitude. The above observations confirmed the formation of channels in the PEI-ICM that are capable of selectively transporting monovalent ions.

### Single-ion transport property in PEI-ICM

To achieve high ion-selective transport efficiency in the PEI-ICM, we regulated the UV-W process time to optimize the channel size of the membrane (Fig. S16). The K^+^/Mg^2+^, Na^+^/Mg^2+^ and Li^+^/Mg^2+^ selectivities of the PEI-ICM with 10 h of UV-W process were 2209, 1603 and 1247, respectively, which were twice those of PEI-ICM with 20 h of UV-W process (Fig. 4b). In contrast, the K^+^, Na^+^ and Li^+^ conductance of the PEI-ICM more than doubled as the UV exposure time increased to 20 h (Fig. 4b, inset). These results further confirmed the precise modulation of membrane channels at the angstrom scale through the UV-W process. As the UV exposure time increased to 30 and 40 h, the selectivity of the PEI-ICM sharply decreased to below 4, while the corresponding ion conductance only slightly increased. Notably, the PEI-ICM with 20 h of UV-W process exhibited excellent ion transport performance, which contradicts the channel size obtained from PALS characterization. This apparent discrepancy arises from dehydration-induced shrinkage of hydrophilic channels during vacuum-based PALS testing (Fig. S17). Accordingly, we chose 20 h of UV-W-processed PEI-ICM, which balanced selectivity and conductance, as the primary object for subsequent tests.

We then explored the relationship between the PEI-ICM conductance and ion concentration by varying salt (KCl, LiCl, CaCl_2_, MgCl_2_) concentrations from 0.001 to 2.0 M (Fig. 4c, Fig S18a–g). As shown in Fig. 4c, the K^+^ and Li^+^ conductance increased steadily between 0.01 and 0.5 M, then plateaued due to ion saturation in the channels (Fig. S18h). Moreover, the Ca^2+^ and Mg^2+^ conductance increased slowly and linearly with ion concentration (Fig. S18i), resulting in the following conductance order: K^+^ > Li^+^ >> Ca^2+^ > Mg^2+^. The K^+^/Mg^2+^ selectivity of the PEI-ICM increased from 147 to 1539 as the concentration rose from 0.01 to 1 M, and then slightly decreased to 1259 as the concentration was further increased to 2.0 M (Fig. 4d). The considerable ion selectivity of the PEI-ICM at high concentrations makes it suitable for high-salt-concentration separation environments.

Besides angstrom-sized channels, the UV-W process generates abundant oxygen-containing functional groups, such as carboxyl groups, which impart tunable charges within the channels and influence ion transport. Thus, we investigated the pH dependence of ion-selective transport (Fig. S19). As solution pH increased from 2 to 8, KCl conductance rose markedly, while MgCl_2_ conductance dropped significantly, increasing K^+^/Mg^2+^ selectivity from 8 to 1173 (Fig. 4e). This behavior is attributed to the deprotonation of carboxyl groups at higher pH, leading to increased negative surface charge of ion channels (Fig. S20). The zeta potential confirmed this trend, reaching −45 mV at neutral pH, indicating substantial carboxyl group ionization on the membrane surface and within its channels, which enables pH-tunable ion selectivity of the membrane.

Additionally, the PEI-ICM showed conductance values in the following order: KCl > NaCl > LiCl >> CaCl_2_ > MgCl_2_ > LaCl_3_ at all voltages (Fig. S21a), which is distinct from those of the PEI chemical etching membrane (PEI-CEM) with nanometer-size channels (Fig. S22a and b). In particular, considering the significant size difference between the two membrane channels, the monovalent metal ion conductance of the PEI-ICM was only about an order of magnitude lower than that of the PEI-CEM (Fig. S22c and d). The ion selectivity of the PEI-ICM at 1, 2 and 5 V exhibited no visible changes with variations of applied voltage (Fig. 4f, Fig. S21b), with the selectivities of K^+^/Mg^2+^, Na^+^/Mg^2+^, Li^+^/Mg^2+^, Ba^2+^/Mg^2+^, Ca^2+^/Mg^2+^ and La^3+^/Mg^2+^ being around 1113, 1024, 644, 1.24, 1.16 and 0.96, respectively. These results demonstrated that the PEI-ICM with highly charged angstrom-sized channels exhibits outstanding monovalent/divalent ion separation performance.

Apart from ion conductance tests, we also conducted electrodialysis diffusion tests to further study the ion-selective separation of the PEI-ICM (Fig. S23). The PEI-ICM showed high permeation rates for K^+^, Na^+^ and Li^+^ of 0.52, 0.45 and 0.24 mol m^−2^ h^−1^ at 1 V, respectively, and these increased to 3.0, 2.5 and 1.7 mol m^−2^ h^−1^ at 5 V (Fig. 4g, Table S2). The PEI-ICM showed a sharp size-exclusion cut-off of ∼7.8 Å at all voltages. The cut-off effect of ion transport in the PEI-ICM is closely related to the dehydration energy barrier of hydrated ions at the channel entrance and the Coulomb interaction between the ions and channel wall charges (Fig. S24, Table S3). This result was confirmed by the ion-activation energy determined by the temperature-dependent ion permeation in the PEI-ICM (Fig. 4h). Moreover, we tested the ion conductance of the PEI-ICM in quaternary ammonium chloride solutions (Fig. S25). For the quaternary ammonium ions with identical charge, the ion conductance sharply decreased with an increase in ion diameter, and similarly exhibited a size-exclusion cut-off at ∼7.8 Å (Fig. S26).

Furthermore, we studied the ion-selective transport in the PEI-ICM by calculating the cation/Cl^−^ mobility ratio through drift-diffusion experiments (Fig. S27). In marked contrast to the PEI-CEM and bulk water, the mobility ratio changed by two orders of magnitude, with an increasing hydrated diameter from K^+^ to La^3+^, with a visible size-exclusion cut-off also existing at ∼7.8 Å (Fig. 4i). This cut-off behavior does not entirely follow the profile of ion hydration energy observed in other angstrom-sized channels, such as angstrom-scale slits, indicating the indispensable effect of electrostatic interactions for ion-selective transport in the highly charged membrane channel.

### Ion separation performance in PEI-ICM

**Figure 5.**
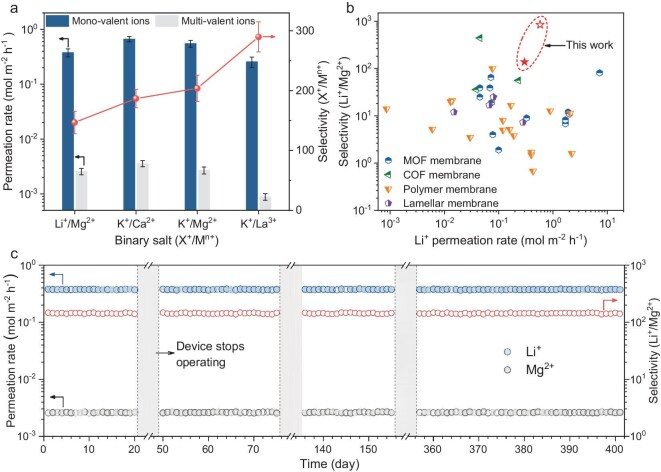
Ion separation and Li extraction performance of membranes. (a) Ion permeation and selectivity performance of PEI-ICM in binary salt systems. (b) Comparison of the Li^+^/Mg^2+^ separation performance of state-of-the-art ion-separation membranes. The symbols represent ion separation in the single (open) and mixed (solid) salt solution of PEI-ICM. (c) Li^+^ and Mg^2+^ permeation rates and Li^+^/Mg^2+^ selectivity of PEI-ICM during long-term separation of mixed salts.

After the single-ion transport behavior, we evaluated the membrane separation performance in binary salt solutions (Fig. S28, Table S4) to explore its applications in Li extraction from saline brine. The binary Li^+^/Mg^2+^, K^+^/Ca^2+^, K^+^/Mg^2+^ and K^+^/La^3+^ selectivities of the PEI-ICM, which are an order of magnitude higher than conventional polymer membranes, were up to 147, 187, 204 and 290, respectively, with the permeation rate of the mono-ion, which is comparable to those of state-of-the-art polymer membranes, reaching 0.3 mol m^−2^ h^−1^ (Fig. 5a and b, Table S5). Meanwhile, the PEI-ICM shows excellent performance compared to emerging membrane materials, including MOFs, COFs and lamellar membranes. Additionally, the PEI-ICM also exhibits outstanding mono-/multivalent ion separation performance during several hundred days of continuous operation, revealing excellent chemical and structural stability (Fig. 5c, Fig. S29).

For practical ion separation applications, we prepared simulated solutions according to the mass ratios of Mg to Li (R_Mg/Li_) in natural brine water as feed solutions to perform electrodialysis Li extraction (Table S6). The membranes showed Li^+^ selectivity even at a high R_Mg/Li_. Under the voltage of 1 V, when the R_Mg/Li_ of feed solution was 20, the R_Mg/Li_ of the solution reduced to 1.8 after the separation of the PEI-ICM, and when the R_Mg/Li_ of feed solution was increased to 60, the R_Mg/Li_ of the solution decreased to 5.8 after the separation. The result indicates that the PEI-ICM has excellent Li extraction performance and repeatability (Figs S30 and S31). Compared to existing commercial membranes, the PEI-ICM exhibits promising performance for Li extraction (Table S7). It can be anticipated that high-purity Li can be extracted from low-Li brine water achieved by simply employing a two-stage membrane series.

## CONCLUSIONS

We propose a UV-water induced chemistry approach to creating angstrom-sized channels in polymer membranes. By regulating the UV irradiation conditions, hydroxyl radicals can scissor out channels with various sizes at the angstrom scale. The resulting channels enable precise control over the transport of monovalent ions over divalent ions, thereby exhibiting higher ion separation efficiency than other polymer membranes. These membranes exhibit excellent structural strength and flexibility, offering the potential for efficient and scalable fabrication through mature manufacturing processes. Our strategy could open up a new avenue for constructing angstrom-scale channels in organic materials widely involved in fuel cells, spent fuel reprocessing and biomimetic transport processes.

## METHODS

### Polymer ion channel membrane fabrication via the UV-W process

To illustrate the UV-W process, angstrom-sized channels were introduced into several different types of polymer membranes (including PEI-IIM, PI-IIM, PET-IIM) to form polymer ion channel membranes (P-ICMs), by this approach. First, the ion-irradiated membrane was completely immersed in a glass Petri dish filled with deionized water. Each side of the membrane was then perpendicularly exposed to UV radiation (MUA-165, Mejiro Genossen, Japan) with a peak wavelength of 365 nm and a power density of 35 mW cm^−2^ at room temperature, with the light power being calibrated by a UV irradiance meter (Model UV-A, China). The UV exposure times ranged from 10 to 40 h for PEI-IIM to form PEI-ICM, 10 h for PI-IIM to form PI-ICM and 2 h for PET-IIM to form PET-ICM, depending on the difference in UV tolerance of membranes. The resulting P-ICM was rinsed with deionized water and preserved in deionized water until experimental testing.

## Supplementary Material

nwaf404_Supplemental_File
