# Supplementary material for: Ultraviolet-water-induced angstrom-sized channels in membrane for precise ion sieving
Source: Natl Sci Rev. 2025 Sep 22;12(11):nwaf404. doi: 10.1093/nsr/nwaf404 (PMC12596271; doi:10.1093/nsr/nwaf404)
Supplement: nwaf404_Supplemental_File [file nwaf404_supplemental_file.pdf]

Supplementary Information

Ultraviolet-water induced angstrom-sized channels in membrane for precise ion sieving

Yaxiong Cheng[1†], Baochun Meng[1†], Huijun Yao[2], Haijian Shi[1], Guozhen Liu[1], Guining Chen[1], Gongping Liu[1,3]*, Wanqin Jin[1]*, Nanping Xu[3]

[1]*State Key Laboratory of Materials-Oriented Chemical Engineering, College of Chemical Engineering, Nanjing Tech University, Nanjing, 211816, China.*

[2]*Institute of Modern Physics, Chinese Academy of Sciences, Lanzhou 730000, P. R. China*

[3]*Suzhou Laboratory, Suzhou 215125, China*

[†]These authors contributed equally to this work.

[*]Corresponding authors: gpliu@njtech.edu.cn (Prof. G. Liu); wqjin@njtech.edu.cn (Prof. W. Jin)

Content

1. Supplementary Methods 3
1.1 Materials 3
1.2 Polymer ion irradiation membrane fabrication via ion irradiation technique 3
1.3 Polymer swelling channel membrane fabrication 4
1.4 Polymer chemical etching membrane fabrication 4
1.5 Membrane characterizations 4
1.6 Gas permeance measurements 6
1.7 Ion transport measurements 6
1.8 Ion permeation experiments 7
1.9 Ion mobility ratio in membrane 9
2. Supplementary Figures 10
2.1 Ion track formation mechanism (Fig. S1) 10
2.2 Preparation of water passages in polymer membranes (Fig. S2) 11
2.3 Monte Carlo simulation of swift heavy ions irradiation on PEI membranes (Fig. S3) 12
2.4 Characterization of physicochemical properties of PEI-based membranes (Figs. S4-S9) 13
2.5 Chemical reactions of polymer membranes during UV-W process (Fig. S10) 19
2.6 Schematic of the preparation of PES-SCM (Fig. S11) 20
2.7 Universality tests of UV-W strategy (Figs. S12-S13) 21
2.8 Ion transport property tests of PEI-ICM (Figs. S14-S27) 23
2.9 Ion separation performance of PEI-ICM (Figs. S28-S31) 37
3. Supplementary Tables 41
4. References 49

1. Supplementary Methods

1.1 Materials

Polyetherimide (PEI, Ultem® 1000, 10 μm thick), polyethylene terephthalate (PET, Lumirror®, 10 μm thick), polyimide (PI, Kapton® HN, 10 μm thick) and polyethersulfone (PES, Ultrason® E, 25 μm thick) films were purchased from Goodfellow Cambridge. Most of the chemicals were purchased from Sigma-Aldrich and Macklin, including potassium chloride (KCl, >99%), sodium chloride (NaCl, >99%), lithium chloride (LiCl, >99%), magnesium chloride ($MgCl_2$, >98%), calcium chloride ($CaCl_2$, >97%), barium chloride ($BaCl_2$, >99%), hexahydrate lanthanum chloride ($LaCl_3 \cdot 6H_2O$, >99%), concentrated hydrochloric acid (HCl, 37%), potassium hydroxide (KOH, >90%), sodium hypochlorite solution (NaClO, ~5%), potassium iodide (KI, >99%), ammonium chloride (H_4NCl, >99%), tetramethylammonium chloride ($(Me)_4NCl$, >98%), tetraethylammonium chloride ($(Et)_4NCl$, >98%), tetrapropylammonium chloride ($(n\text{-}Pr)_4NCl$, >98%), tetrabutylammonium chloride ($(n\text{-}Bu)_4NCl$, >97%), tetrapentylammonium chloride ($(n\text{-}Pe)_4NCl$, >98%), dimethyl sulfoxide (DMSO, ≥99.9%). The deionized water was made in laboratory. All chemicals were used without further purification.

1.2 Polymer ion irradiation membrane fabrication via ion irradiation technique

Polymer membranes (including PEI, PI and PET) were clamped into customized sample holder before irradiation. Samples were then loaded into vacuum sample chamber with their surface perpendicular to the beam direction. Swift heavy ion beam provided by HIRFL (Heavy Ion Research Facility in Lanzhou) was uniformly dispersed by two scanning magnets (including X and Y direction), and the ion fluence was monitored with a secondary electron detector. The statistical error of detector counting typically is <5%. Polymer membranes were irradiated with 2.15 GeV $^{86}Kr^{26+}$ ions to form polymer ion track membrane (P-IIM) with narrow and continuous ion tracks. Those ion tracks consist of well-defined free volumes across membranes. The irradiation fluence was 1×10^{10} ions cm^{-2} and

5×10^{10} ions cm^{-2}, with the relatively low fluence samples being used to fabricate nanochannels. The irradiation density of membranes was calibrated with the density of the nanopores made of the track etching technique, and it was confirmed to be consistent with the irradiation fluence.

1.3 Polymer swelling channel membrane fabrication

Polymer membranes can swell in organic solvents to generate continuous free volumes in their polymer networks, forming polymer swelling membranes (P-SMs). To demonstrate the solvent swelling approach, polyethersulfone (PES) membrane was used as an example, which can undergo swelling in dimethyl sulfoxide (DMSO) solution. The solvent used for swelling PES membrane was prepared by mixing DMSO and deionized water in a volume ratio of 5: 2. The PES membrane was immersed in the mixed solution for swelling, lasting for about 2 h. The resulting PES-SM was thoroughly cleaned with deionized water and was soaked in deionized water for storage. Afterward, each side of the PES-SM was perpendicularly exposed to UV radiation for 1 h to form PES swelling channel membrane (PES-SCM).

1.4 Polymer chemical etching membrane fabrication

To create nanochannel in membrane, we used PEI-IIM irradiated with 2.15 GeV $^{86}Kr^{26+}$ ions in a fluence of 1×10^{10} ions cm^{-2} as a template. NaClO solution with a mass fraction of 5% was used as an etchant to chemically etch the ion tracks within PEI-IIM. To obtain uniform cylindrical nanochannels, the PEI-IIM was chemically etched for 8 min by soaking the membrane in the NaClO etchant solution at 50 °C with ultrasonic assistance. The cylindrical track-etched nanochannels can be generated in the PEI-IIM to form PEI chemical etching membrane (PEI-CEM). Finally, the etched membrane was rinsed in deionized water three times to clean the residual etchant and dried in air.

1.5 Membrane characterizations

The surface morphologies and cross-sectional structures of the membranes were observed using field-emission scanning electron microscopy (FESEM, Hitachi S-4800, Japan). Before testing, the

membranes were manually fractured in liquid nitrogen and sputter-coated with a thin layer of platinum to inhibit the charging effect. The surface roughness and three-dimensional topography of the membranes was measured using atomic force microscope (AFM, Bruker Dimension® Icon, Germany). The stress-strain curve of the membrane was subjected to tensile testing using a dynamic mechanical analyzer (Discovery DMA850, TA, America), and the samples size of the membrane was 5 mm × 15 mm. The evolution of chemical composition in the membranes was analyzed by ^{1}H nuclear magnetic resonance (^{1}H NMR, Bruker 400 MHz, Germany), attenuated total reflectance Fourier transform infrared spectrometer (ATR-FTIR, PerkinElmer Spectrum IR, America) and X-ray photoelectron spectrometer (XPS, Thermo Scientific K-Alpha, America). Membrane samples for NMR testing were dissolved in DMSO-d_6 by heating to 180 ℃ before testing. To eliminate the noise from adsorbed water molecules on hydroxyl group detection in ATR-FTIR test, membrane samples were vacuum-dried at 50 °C for 48 h before testing. The C–C characteristic peak at 284.8 eV was used as a charge correction reference for the XPS analyses. Thermal and pyrolysis product analyses were performed using thermogravimetric-mass spectrometry (TG-MS, SDT 650+Discovery MS, America). Membrane samples were heated from 25 to 800 °C under argon purging at a heating rate of 10 °C min^{-1}. The surface zeta potentials of the membranes were measured using electro-kinetic analyzer (Anton Paar Surpass 3, Austria) with an adjustable gap cell. The free volume size and channel radius in the membranes were determined using positron annihilation lifetime spectroscopy (PALS, DPLS3000, China) with a time resolution of 210 ps and Doppler broadening energy spectroscopy (DBES) with a variable monoenergy slow positron beam (0.18-10.18 keV). This radioisotope beam used 20 μCi ^{22}Na as the positron source. A contact angle meter (CA, Attension Theta Lite, Sweden) was used to determine the water contact angle on the membrane surface. Ion distribution in the membrane channels was detected using field emission transmission electron microscope (FETEM, FEI Talos F200X G2, America) equipped with energy-dispersive X-ray

spectroscopy (EDX) at an acceleration voltage of 200 kV. Samples were prepared as follows: clean the surface of the membrane soaked in salt solutions with deionized water, and slice the membrane embedded with Spurr low viscosity embedding agent using ultramicrotome (Leica EM FC7, Germany) to get samples. The hydroxyl radicals (·OH) stemming from UV-W process were detected using electron paramagnetic resonance (EPR, Bruker A200, America). Samples were prepared by exposing deionized water to UV light for 20 h, then added 5,5-dimethyl-1-pyrroline N-oxide (DMPO) as the hydroxyl radical scavenger to the water and measured the EPR of the water sample within 5 min.

1.6 Gas permeance measurements

The gas permeance of single-component gases was measured using the constant volume permeation system. The feed pressure and temperature were set at 1 bar and 30 °C, respectively. The permeation measurements of each gas were performed at least three times after the system reached a steady state. The gas permeance can be calculated using the following equation:

$$P = \frac{273.15}{273.15 + T} \times \frac{V_m}{A} \times \frac{l}{\Delta p} \times \frac{dp}{dt} \quad (1)$$

where P is the gas permeance (Barrer, 1Barrer = 10^{-10} cm^3(STP)cm cm^{-2} s^{-1} $cmHg^{-1}$), T is the permeation temperature (°C), V_m is the downstream volume (cm^3), A is the effective membrane area (cm^2), Δp is the transmembrane pressure (cmHg).

1.7 Ion transport measurements

Ion transport properties of membranes were investigated by current−voltage (I−V) characteristics. Ion currents across the two-chamber Teflon cell separated by the membrane samples were recorded using an electrochemical workstation (CHI 760E, China). Both chambers of the cell were filled with chloride salt solutions, with a pair of Ag/AgCl electrodes in the cell being used to apply electric potential across the studied membrane. The main transmembrane potential used in this work was a scanning voltage that varied from –5 to +5 V. Salt solutions with various concentrations were prepared by diluting the concentrated counterparts. The pH of salt solutions was adjusted using 0.1 M HCl and

0.1 M KOH solutions. Current measurements at all concentrations and pH values were carried out at room temperature. Each test was repeated at least three times to get the average current values at different voltages. The ion and proton transport properties are reflected by the ion conductance (G):

$$G = \frac{I}{U} \tag{2}$$

where the U is the applied transmembrane voltage (V), and the I is the ion current density (A cm^{-2}) measured under the applied transmembrane voltage.

The differences in ion conductance were primarily attributed to the distinct metal ion conduction abilities in the membrane under the same applied voltage, given that the salt solutions all contained the same anion (Cl^-). According to equation (1), the ion selectivity (S) based on ion currents for one metal ions over another ion was calculated by the ratio of the corresponding ion current at the same transmembrane voltage, taking into consideration the valence difference:

$$S_{M^{n+}/X^{m+}} = \frac{I_{MCl_n}}{I_{XCl_m}} \times \frac{m}{n} \tag{3}$$

where the I_{MCl_n} and I_{XCl_m} are the ion current measured in MCl_n and XCl_m solutions, and the m and n are the valence number of the metal ion in salt solution, respectively.

1.8 Ion permeation experiments

Ion permeation across the membranes in single and mixed salt conditions under driving voltage were carried out using stirred H-shaped cells. Membrane samples were clamped between two silicone rings and sealed in the middle of the H-shaped cells. The electrodialysis ion permeation were performed by applying a constant potential (1 V, 2 V and 5 V) via an electrochemical workstation (CHI 760E, China) across the membrane samples using a pair of Pt electrodes. The effective area of the membrane samples in the H-shaped cell was 0.5 cm^2. The magnetic stirring in both feed and permeate solutions aimed to mitigate concentration polarization near the membranes. In single-salt electrodialysis permeation tests, 15 mL of 1 M salt solution (KCl, NaCl, LiCl, $BaCl_2$, $CaCl_2$, $MgCl_2$ and $LaCl_3$) was used as feed solution, and the permeate side was filled with 15 mL deionized water. In binary ion

permeation tests, 15 mL of 1 M mixed salt solution (0.5 M LiCl + 0.5 M $MgCl_2$, 0.5 M NaCl + 0.5 M $MgCl_2$, 0.5 M KCl + 0.5 M $MgCl_2$, 0.5 M KCl + 0.5 M $CaCl_2$, 0.5 M KCl + 0.5 M $LaCl_3$) was used as feed solution, and the permeate side was filled with 15 mL deionized water. In simulated natural brine lake water tests, 15 mL mixed salt solution according to the mass ratios of magnesium to lithium ($R_{Mg/Li}$) in nature brine water as feed solutions ($R_{Mg/Li}$ = 20: 0.1wt% LiCl + 2wt% $MgCl_2$, $R_{Mg/Li}$ = 60: 0.025wt% LiCl + 1.5wt% $MgCl_2$), the permeate side was filled with 15 mL deionized water. The ion concentrations of the permeate solution were measured using inductively coupled plasma optical emission spectrometry (ICP-OES).

In these electrodialysis permeation tests, the permeation rates of ions across a membrane were calculated by the following:

$$P = \frac{\Delta C}{\Delta t} \times \frac{V}{A} \tag{4}$$

where P is the permeation rate; ΔC and Δt are the ion concentration in permeate solution and permeation time, respectively; V is the volume of permeate solution and A is the effective area of the membranes.

The ion selectivity during permeation test can be calculated by the following formula:

$$S_{X^+/M^{n+}} = \frac{P_{X^+}}{P_{M^{n+}}} \tag{5}$$

where $S_{X^+/M^{n+}}$ is the selectivity ratio of X^+ and M^{n+} ions; P_{X^+} and $P_{M^{n+}}$ are the permeation rate of X^+ and M^{n+} ions, respectively.

Meanwhile, the separation factor in simulated natural brine lake water tests can be calculated by the following formula:

$$f = \frac{R_{Mg/Li}}{R^*_{Mg/Li}} \tag{6}$$

where f is the separation factor; $R_{Mg/Li}$ and $R^*_{Mg/Li}$ are the mass ratios of magnesium to lithium in feed solutions and permeate side solutions, respectively.

1.9 Ion mobility ratio in membrane

Drift-diffusion experiments were performed for PEI-ICM and PEI-CEM to gain more information about the influence of channel dimensions on the ion transport. The studied membrane was clamped between two Teflon chambers, with one of the chamber being filled with 1 M chloride salt solution and the other being filled with the same 0.1 M chloride salt solution. Therefore, the concentration gradient of electrolyte (Δ) across the membrane was 10. A scanning voltage ranging from –1 to +1 V was applied with a pair of standard Ag/AgCl glass electrodes filled with saturated KCl solution to avoid diffusion potential. *I*–*V* characteristics for various chloride salt solution were measured to obtain the zero-current potential (E_m), which corresponds to the voltage value at zero current on the *I*–*V* curve. The observed E_m can be explained by the liquid-junction potential arising from the different mobilities of cations and anions within the membrane channels. Based on E_m, the mobility ratio of cations to anions (μ_+/μ_-) be determined by applying the Henderson equation:

$$\mu_+ / \mu_- = -\frac{z_+}{z_-}\frac{\ln(\Delta) - \frac{z_- F E_m}{RT}}{\ln(\Delta) - \frac{z_+ F E_m}{RT}} \quad (7)$$

where z_+ and z_- are the valences of cations and anions, respectively; F is the Faraday constant; R is the universal gas constant; T = 300 K; and Δ is the concentration gradient across the membrane. In our experiments, Δ = 10 and z_- = –1.

2. Supplementary Figures

2.1 Ion track formation mechanism (Fig. S1)

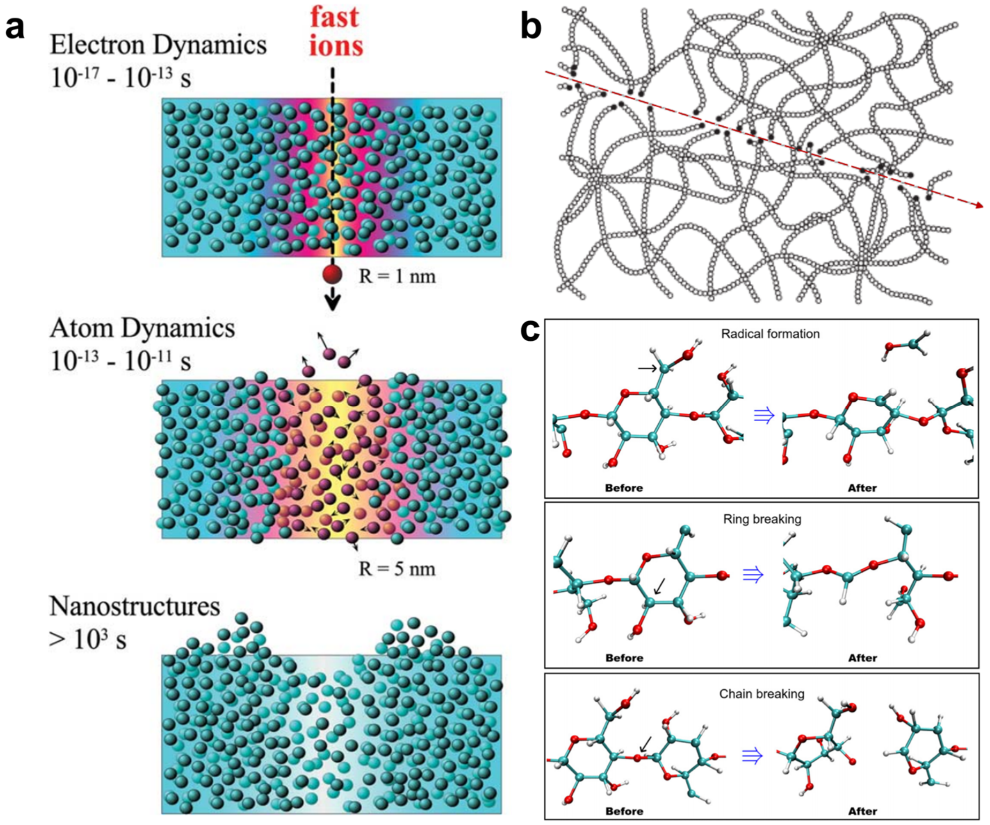

Supplementary Figure 1. (**a**), Time evolution of an ion track. The initial excitation and ionization of atoms induces atomic motions, which freeze out and may lead to permanent rearrangements [1]. (**b**), Heavy ion irradiation of polymer materials. (**c**), Three typical damage types of materials irradiated by fast heavy ions [2].

2.2 Preparation of water passages in polymer membranes (Fig. S2)

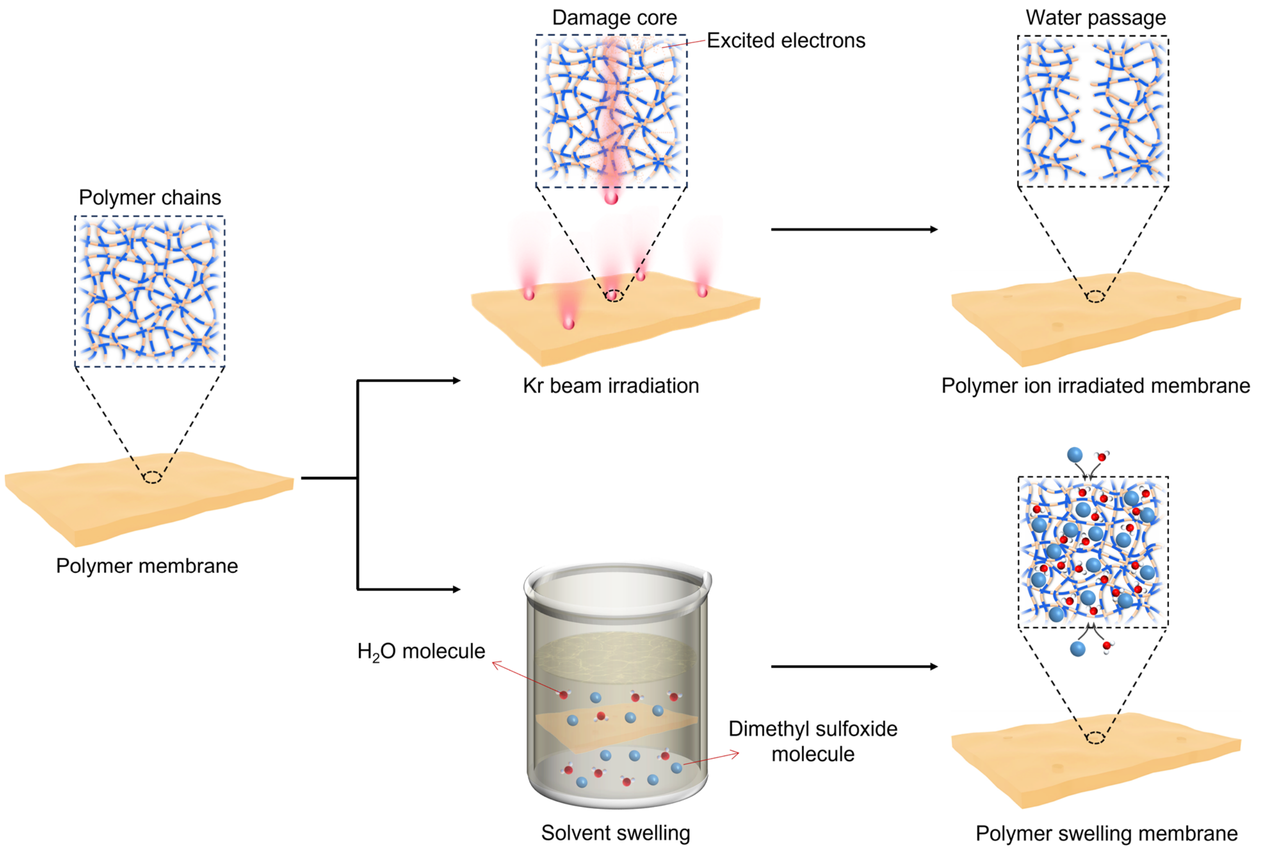

Supplementary Figure 2. Routes of water molecules diffuse into the interior of the polymer membranes. PEI-IIM was prepared by irradiating 10 μm thick PEI membranes with 2.15 GeV $^{86}Kr^{26+}$ beam in a fluence of 5×10^{10} ions cm^{-2}.

2.3 Monte Carlo simulation of swift heavy ions irradiation on PEI membranes (Fig. S3)

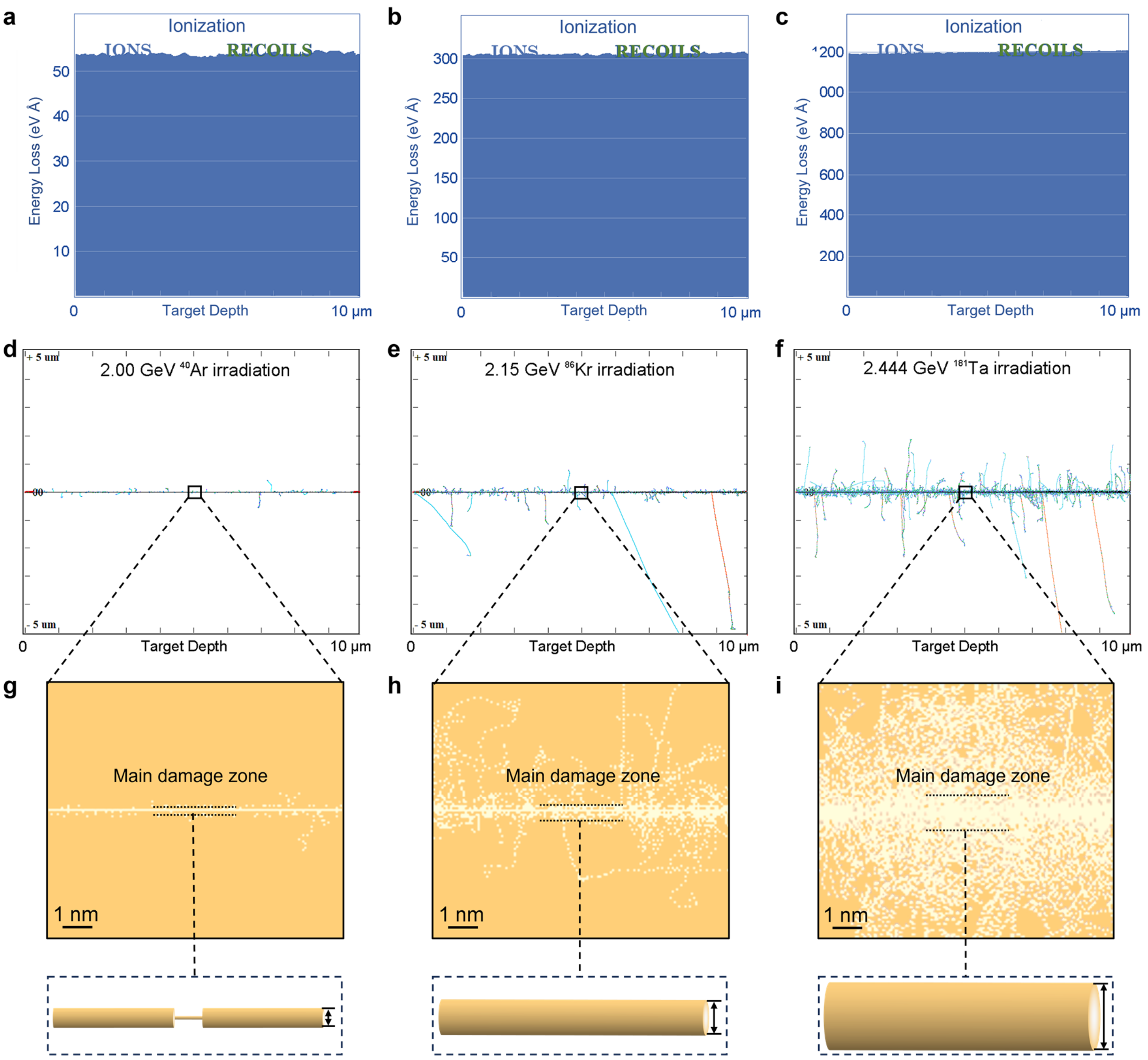

Supplementary Figure 3. (a-c) Energy loss distributions of 2.0 GeV ^{40}Ar (a), 2.15 GeV ^{86}Kr (b) and 2.444 GeV ^{181}Ta (c) in membranes. (d-f) The ion paths and scattering cross sections of (d) 2.0 GeV ^{40}Ar (e) 2.15 GeV ^{86}Kr and (f) 2.444 GeV ^{181}Ta through membranes. The black, red, blue, pink and cyan trajectories represent incident ions, scattered target atoms (C, H, O and N), respectively. (g-i) Ionizing effect induced by (g) 2.0 GeV ^{40}Ar (h) 2.15 GeV ^{86}Kr and (i) 2.444 GeV ^{181}Ta in membranes. The paths composed of white dots represent ionizing events of primary ions (in the center of straight trajectory) and the secondary electrons. The corresponding cylindrical models illustrate the structures of main damage zone. To better illustrate the energy deposition process, these results show the calculation of 1500 ions.

2.4 Characterization of physicochemical properties of PEI-based membranes (Figs. S4-S9)

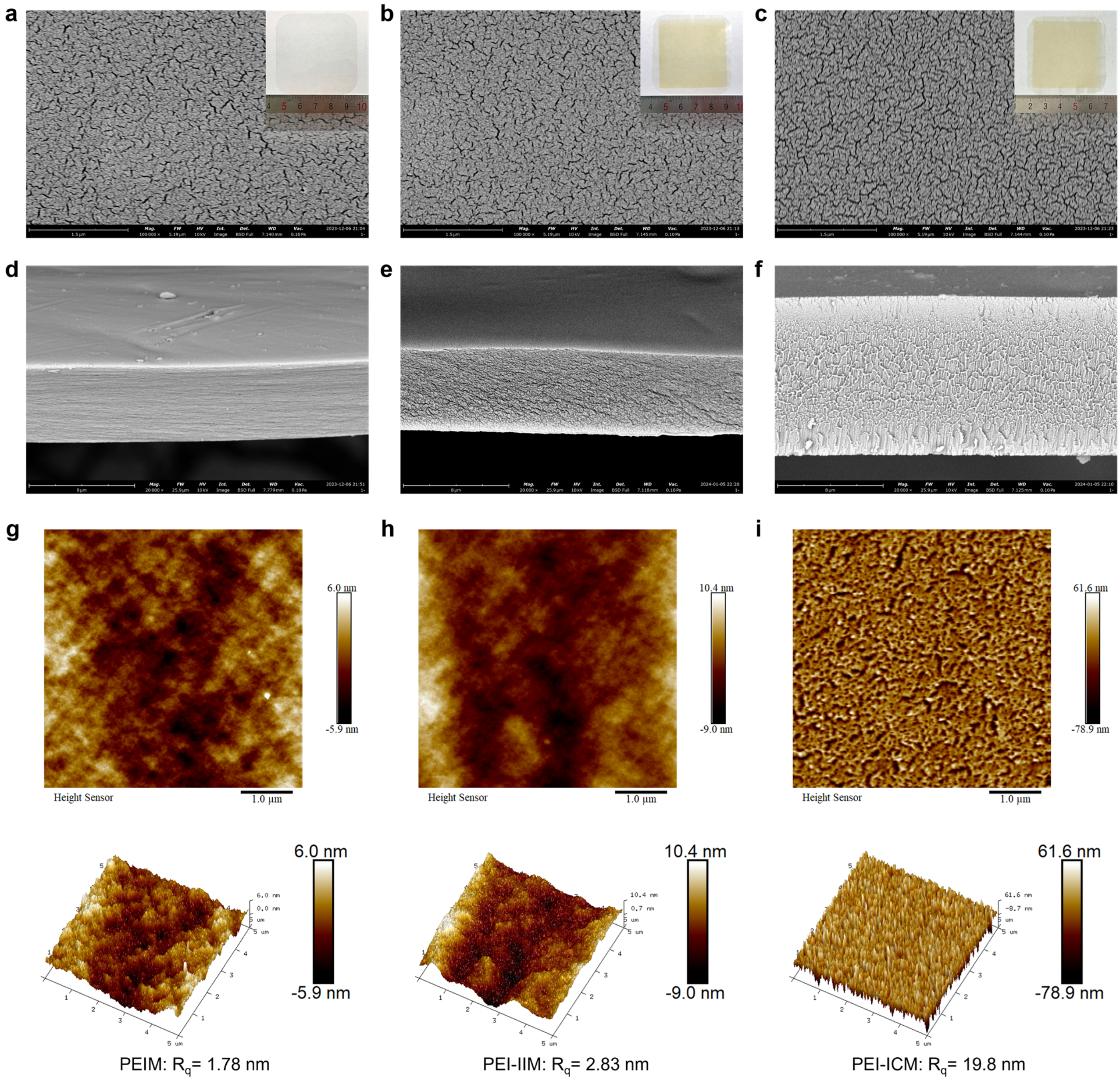

Supplementary Figure 4. Membrane morphologies. (a-c) SEM images of the top surface of the PEIM (a), PEI-IIM (b) and PEI-ICM (c). (Inset) Corresponding digital photos of the PEIM, PEI-IIM and PEI-ICM, respectively. (d-f) Cross-sectional SEM images of the PEIM (d), PEI-IIM (e) and PEI-ICM (f). (g-i) AFM images of the PEIM (g), PEI-IIM (h) and PEI-ICM. (i) Surface roughness (R_q) of the PEIM, PEI-IIM and PEI-ICM are 1.78, 2.83 and 19.8, respectively.

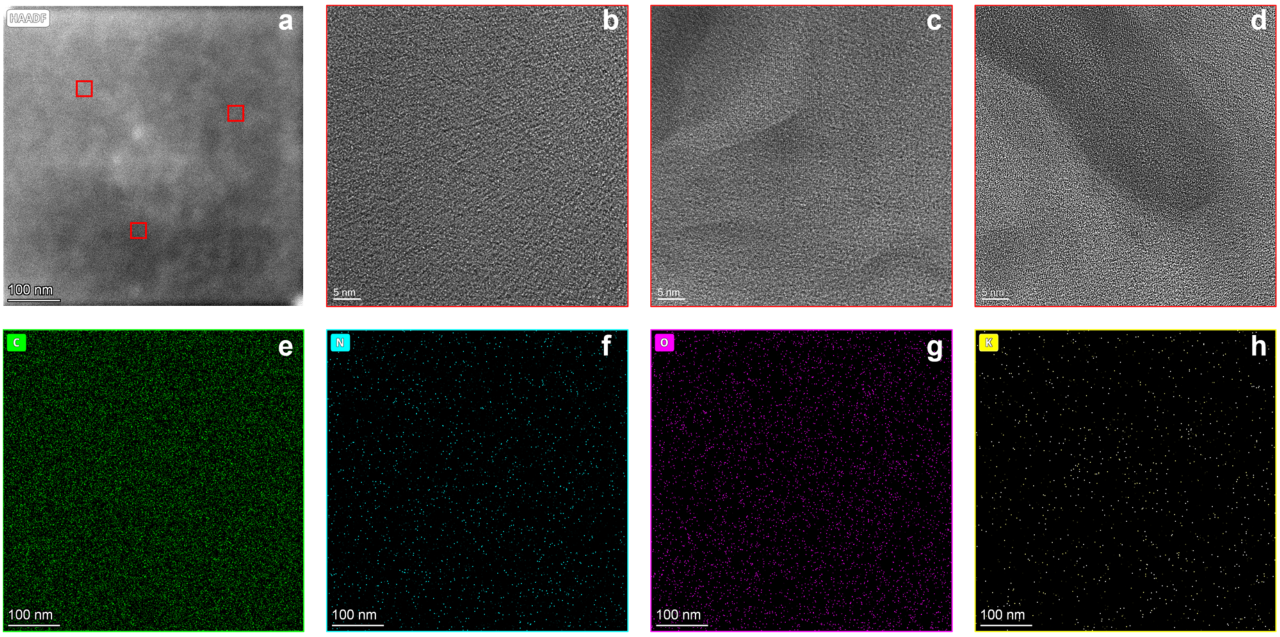

Supplementary Figure 5. TEM image and EDS mapping of PEI-ICM. a, STEM HAADF image of PEI-ICM. (b-d) HRTEM images of the red square area in (a). (e-h) EDX mapping of C (e), N (f), O (g) and permeated K elements (h) of PEI-ICM.

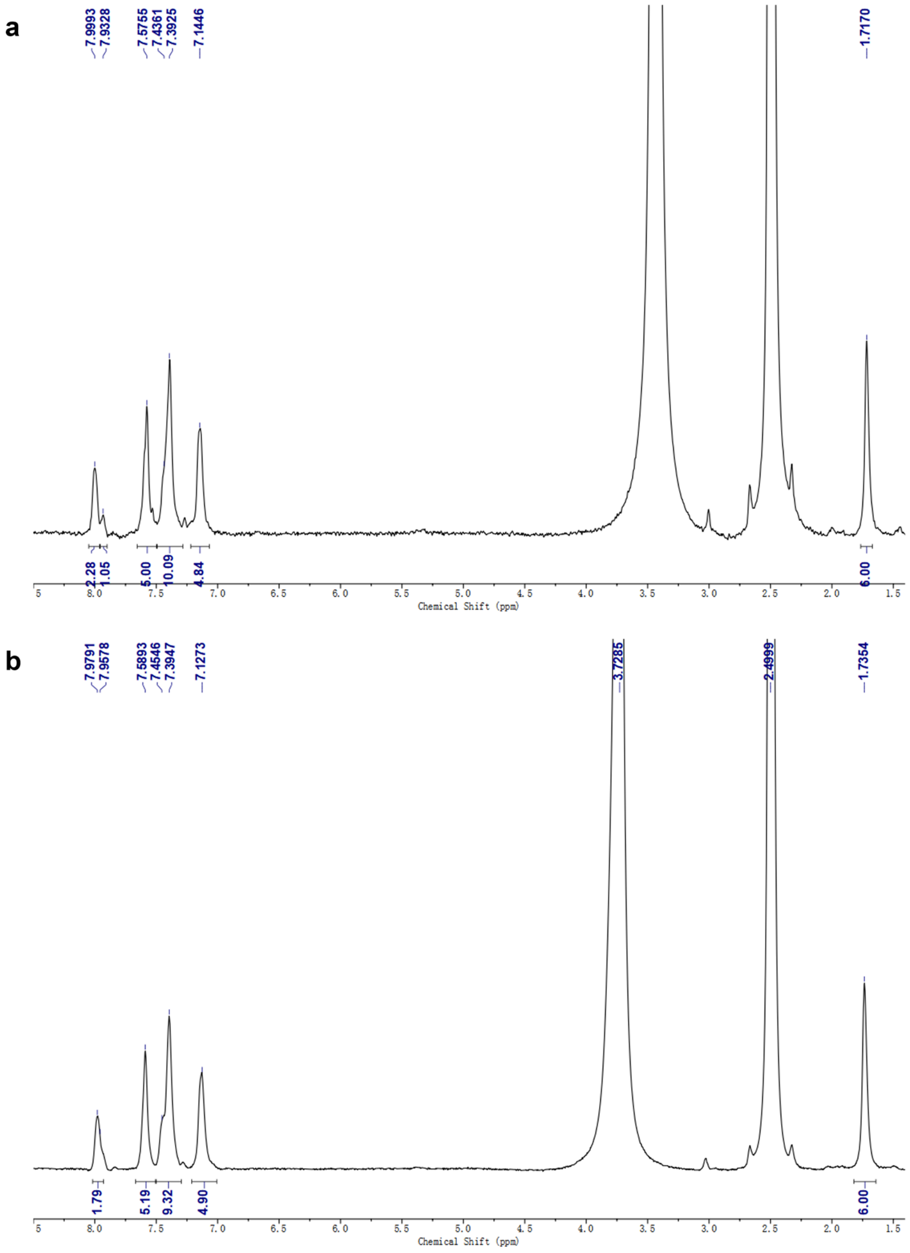

Supplementary Figure 6. ^{1}H NMR (400 MHz, DMSO-d_6) spectra of the PEI-ICM (a) and PEI-IIM (b). a, δ: 8.0 (s, 2H), 7.93 (s, 2H), 7.58 (s, 5H), 7.44~7.39 (m, 10H), 7.14 (s, 5H), 1.72 (s, 6H), b, δ: 7.98~7.96 (m, 2H), 7.59 (s, 5H), 7.45~7.39 (m, 10H), 7.12 (s, 5H), 1.74 (s, 6H)

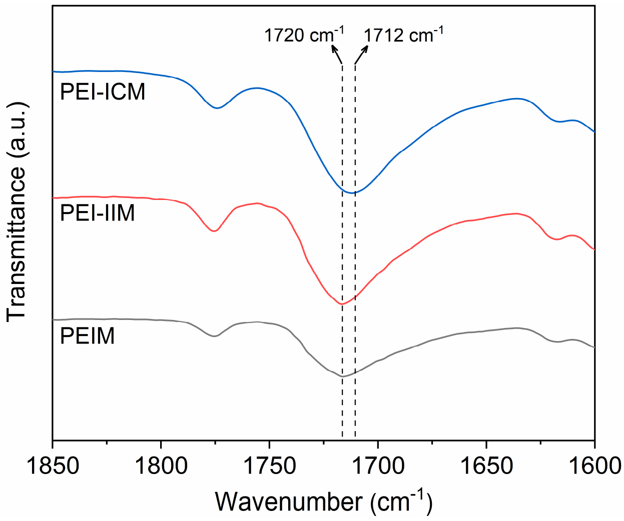

Supplementary Figure 7. FTIR-ATR spectra of the PEIM, PEI-IIM, and PEI-ICM.

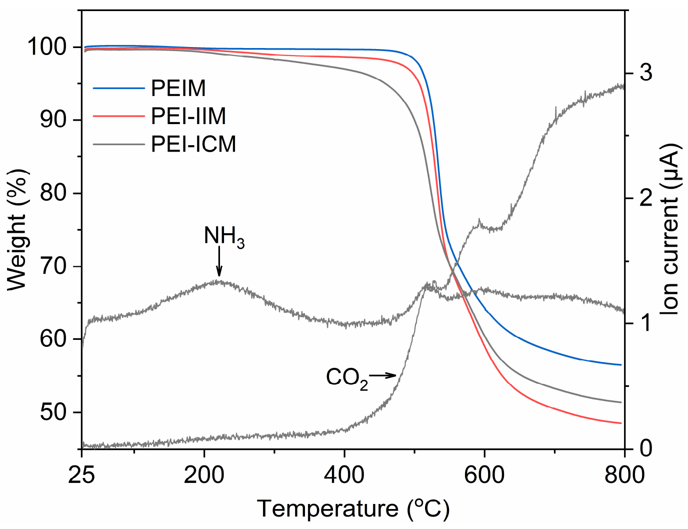

Supplementary Figure 8. TG curves of the PEIM, PEI-IIM, and PEI-ICM and TG-MS curve of the PEI-ICM.

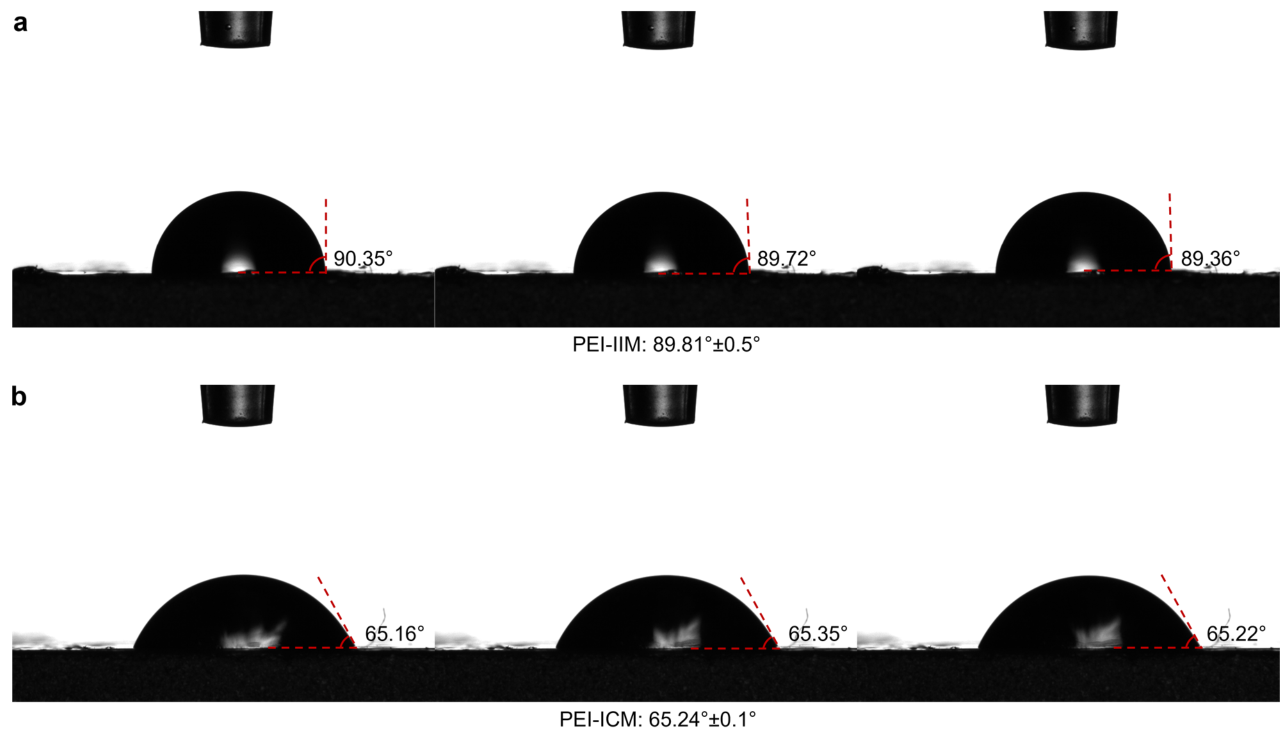

Supplementary Figure 9. Water contact angle of membranes. (a) Water contact angle of PEI-IIM. (b) Water contact angle of PEI-ICM. The membrane transitioned from hydrophobic to hydrophilic after UV-W process.

2.5 Chemical reactions of polymer membranes during UV-W process (Fig. S10)

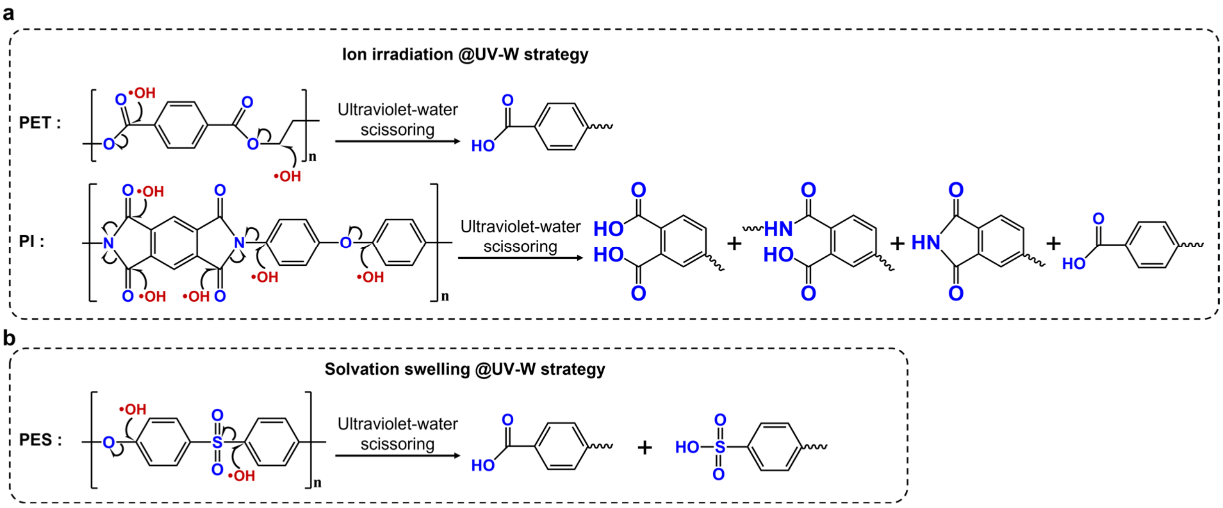

Supplementary Figure 10. (a) The primary chemical reactions for PET and PI during in-situ scissoring with hydroxyl radicals [3, 4]. For PET membranes, hydroxyl radical attack leads to the cleavage of C-O bonds, forming terminal carboxyl groups. For PI membranes, hydroxyl radical attack leads to the cleavage of C-N and C-O bonds, forming terminal carboxyl groups and amino groups. (b) The primary chemical reactions for PES during in-situ scissoring with hydroxyl radicals [5]. For PES membranes, hydroxyl radical attack results in the cleavage of C-O and C-S bonds, forming terminal carboxyl groups and sulfonic acid groups.

2.6 Schematic of the preparation of PES-SCM (Fig. S11)

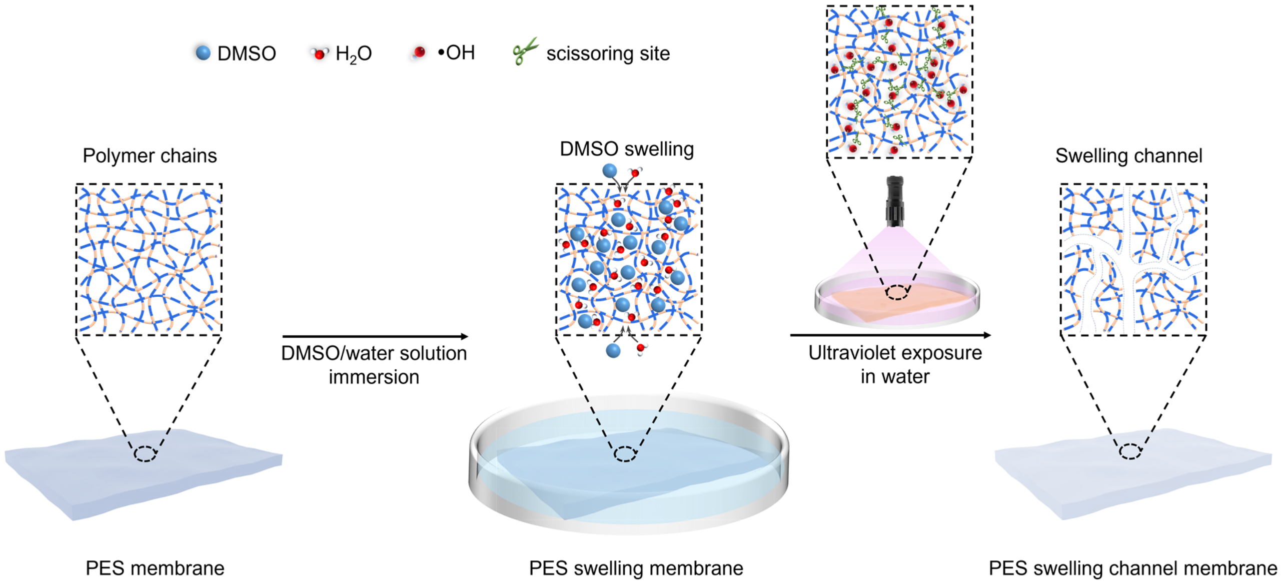

Supplementary Figure 11. Schematic of PES swelling channel membrane (PES-SCM) fabricated by solvation swelling @UV-W process.

2.7 Universality tests of UV-W strategy (Figs. S12-S13)

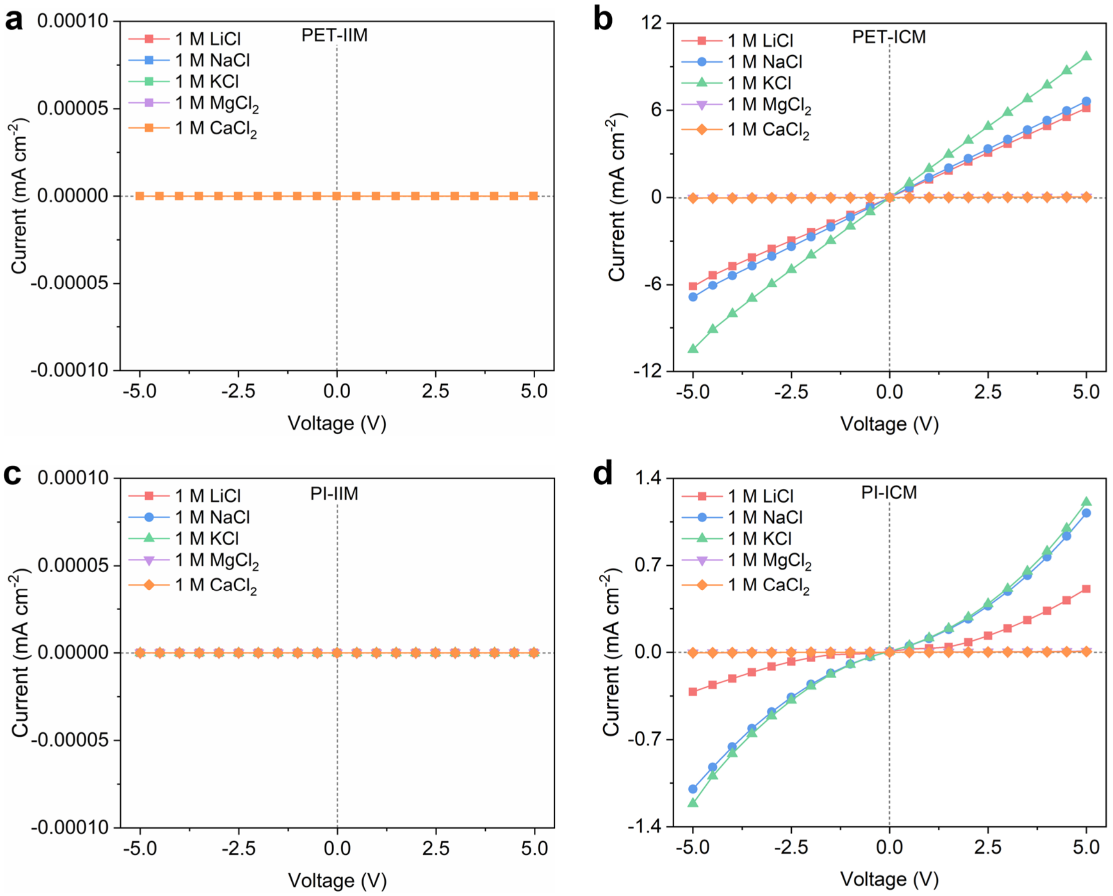

Supplementary Figure 12. Ion transport properties of PET-based and PI-based membranes. (a, b) *I*–*V* curves of PET-IIM (a) and PET-ICM (b). (c, d) *I*–*V* curves of PI-IIM (c) and PI-ICM (d). Both the PET-IIM and PI-IIM were irradiated by 2.15 GeV $^{86}Kr^{26+}$ beam in a fluence of 5×10^{10} ions cm^{-2}. Both the UV-W process time for PEI-ICM and PI-ICM were 2 h.

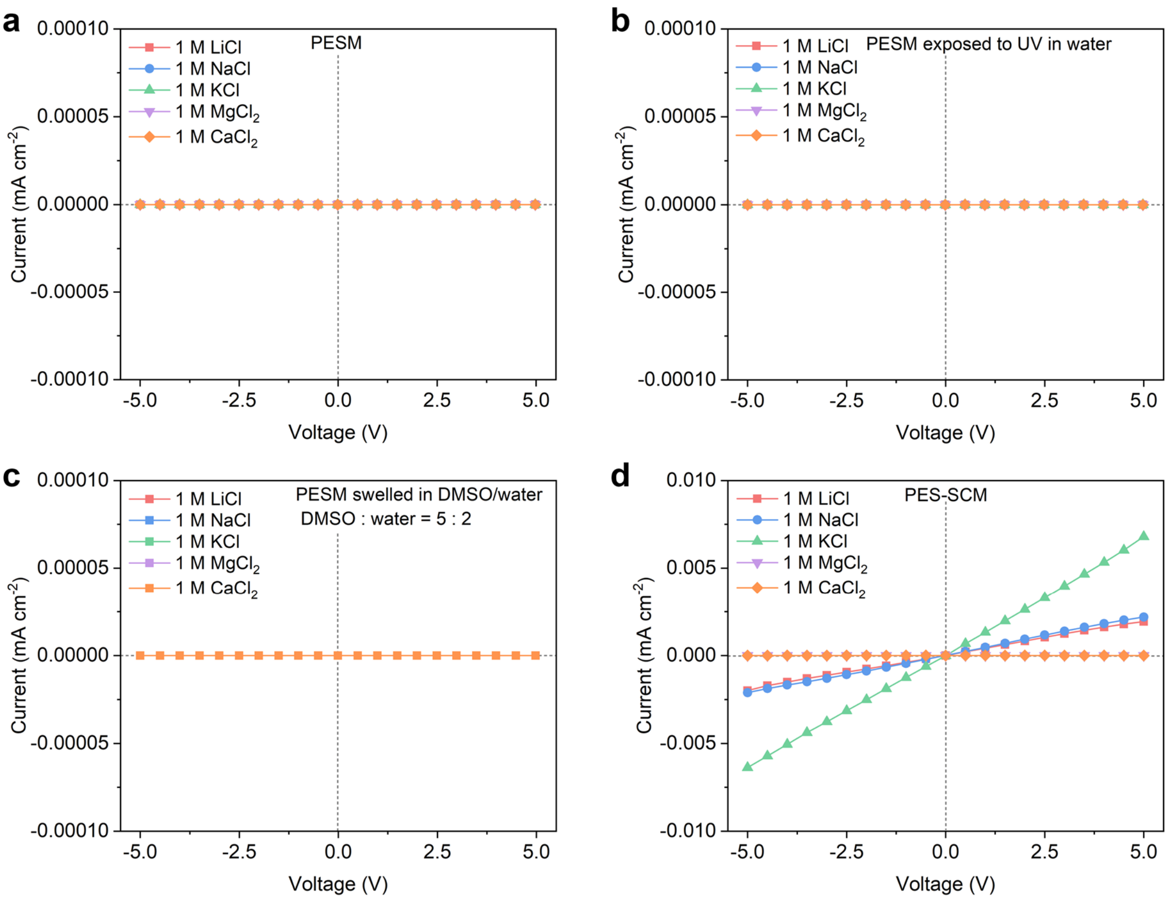

Supplementary Figure 13. Ion transport properties of various PES-based membranes. (a-d) *I–V* curves of pristine PESM (a), PESM exposed to UV light in water for 2 h (b), PESM swelled in DMSO/water mixed solution (DMSO: H_2O = 5: 2) for 2 h (c) and PES-SCM (d), respectively. These results indicate that solvent swelling introduced continuous free volume in PES membranes that only allow the water molecule permeation and subsequent UV-W processing further introduced angstrom-sized channels in these membranes that selectively transport monovalent metal ions.

2.8 Ion transport property tests of PEI-ICM (Figs. S14-S27)

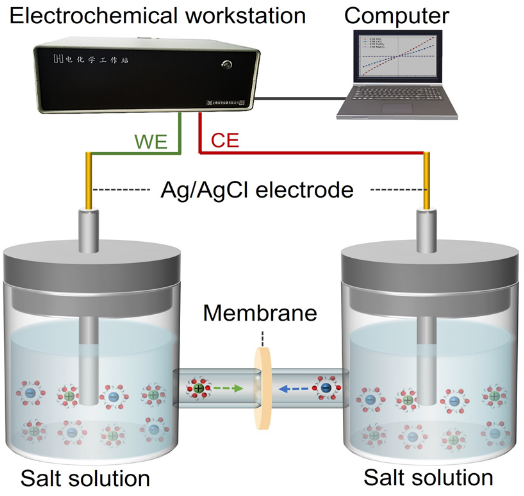

Supplementary Figure 14. Schematic of the current−voltage (*I-V*) curve measurement setup.

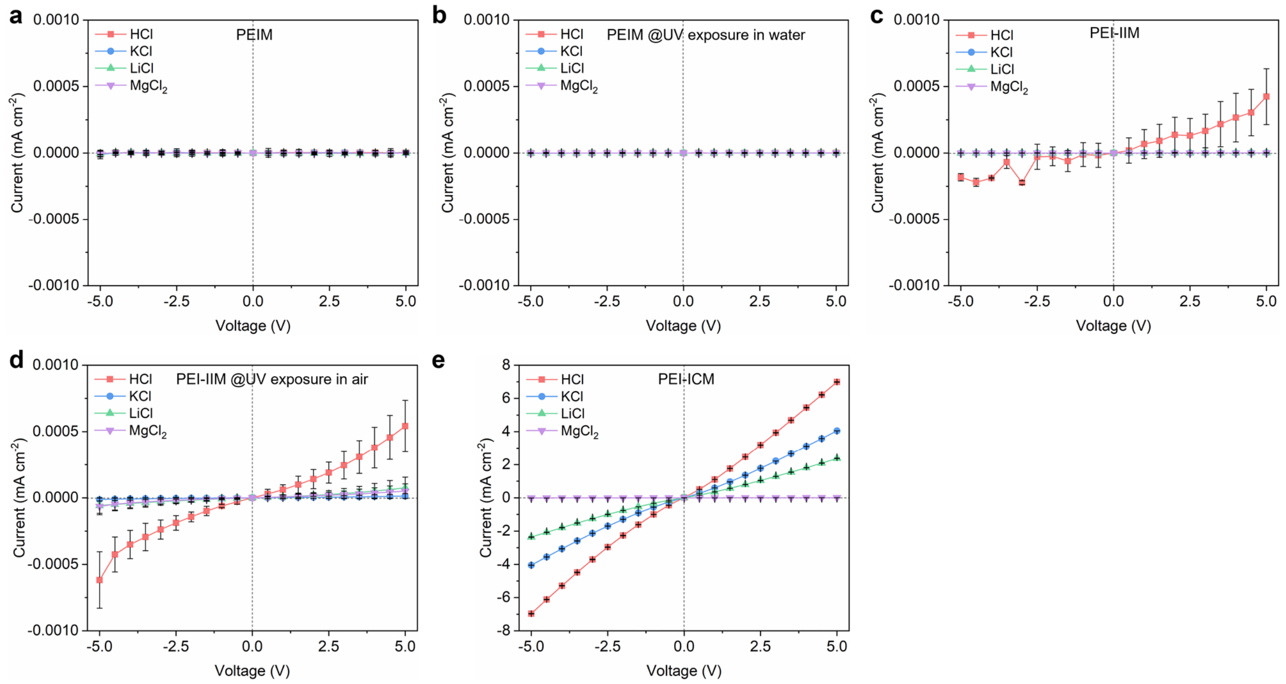

Supplementary Figure 15. Ion transport properties of various PEI-based membranes. (a-e) *I–V* curves of pristine PEIM (a), PEIM exposed to UV light in water (b), PEI-IIM (c), PEI-IIM exposed to UV light in air (d), and PEI-ICM (e), respectively. These results indicate that Kr ion irradiation introduced continuous free volume in PEI membranes that only allow the water molecule permeation and subsequent UV-W process further introduced angstrom-sized channels in these membranes that selectively transport monovalent metal ions.

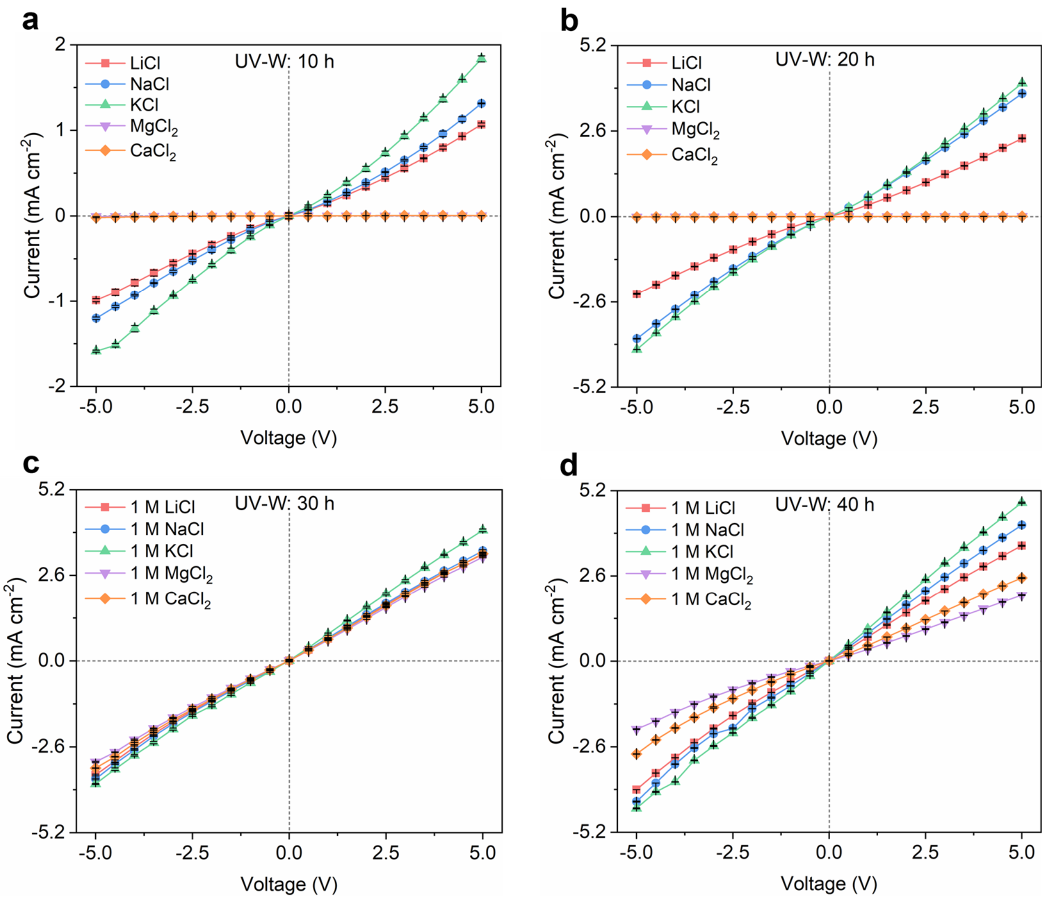

Supplementary Figure 16. Ion selective transport of PEI-ICMs with different UV-W process time. (a-d) *I–V* curves of PEI-ICM subjected to UV-W processing for10 h (a), 20 h (b), 30 h (c), and 40 h (d), respectively.

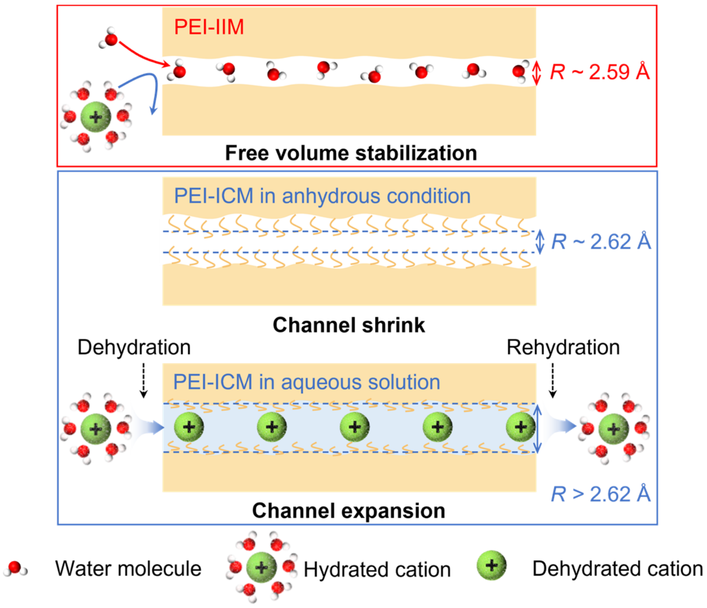

Supplementary Figure 17. Schematic diagrams illustrate the selective transport of hydrated ions and water molecules in the membrane channels of PEI-IIM and PEI-ICM.

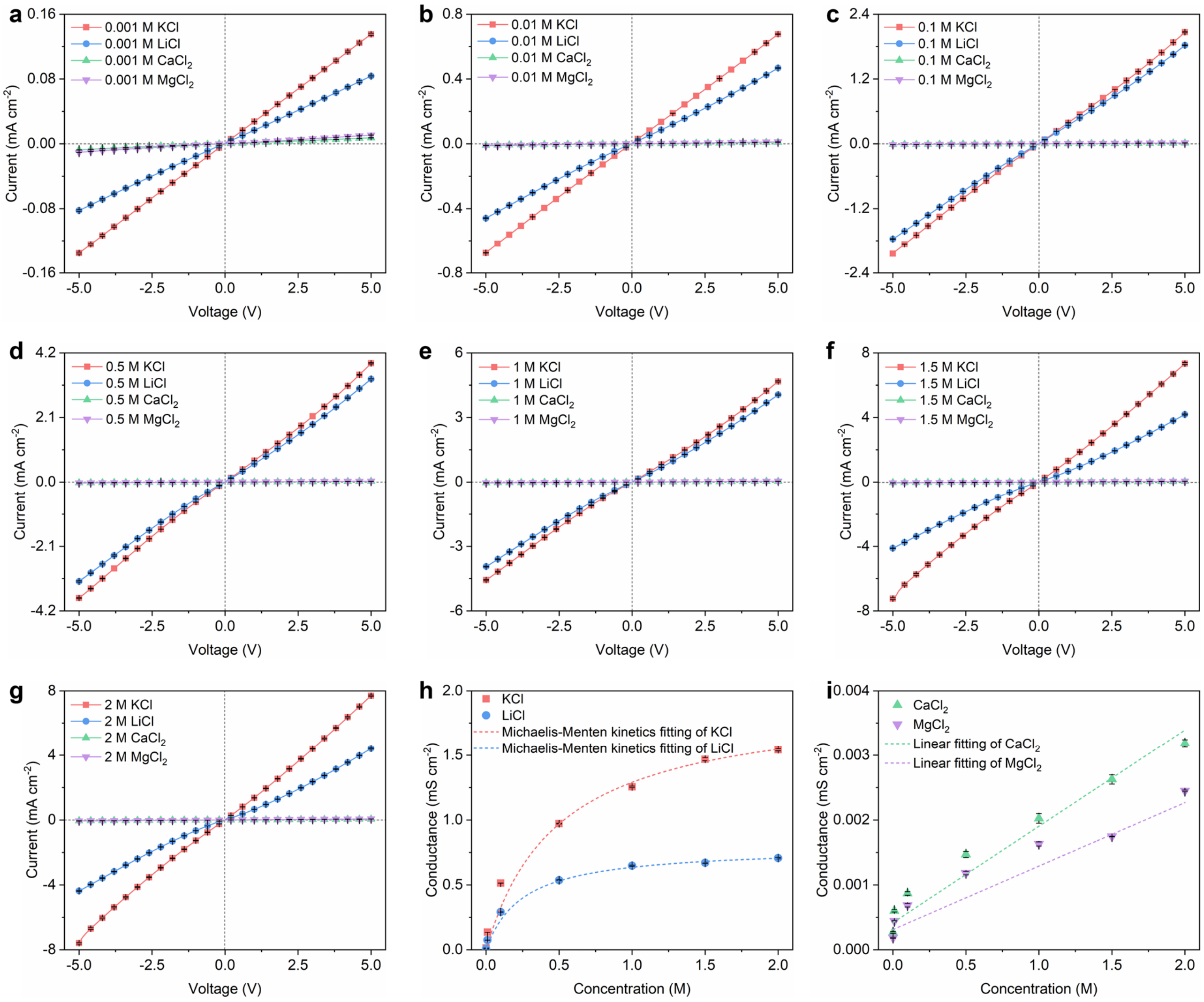

Supplementary Figure 18. Effect of salt concentration on ion-selective transport of PEI-ICM. (a-g) *I–V* curves of PEI-ICM for KCl, LiCl, $CaCl_2$ and $MgCl_2$ with concentration of 0.001 M (a), 0.01 M (b), 0.1 M (c), 0.5 M (d), 1 M (e), 1.5 M (f), and 2 M (g), respectively. h, KCl and LiCl conductance of PEI-TCM as a function of salt concentration. Dashed lines indicate fits to a Michaelis-Menten transport model. i, $CaCl_2$ and $MgCl_2$ conductance of PEI-ICM as a function of salt concentration. Dashed lines indicate fits to a linear transport model.

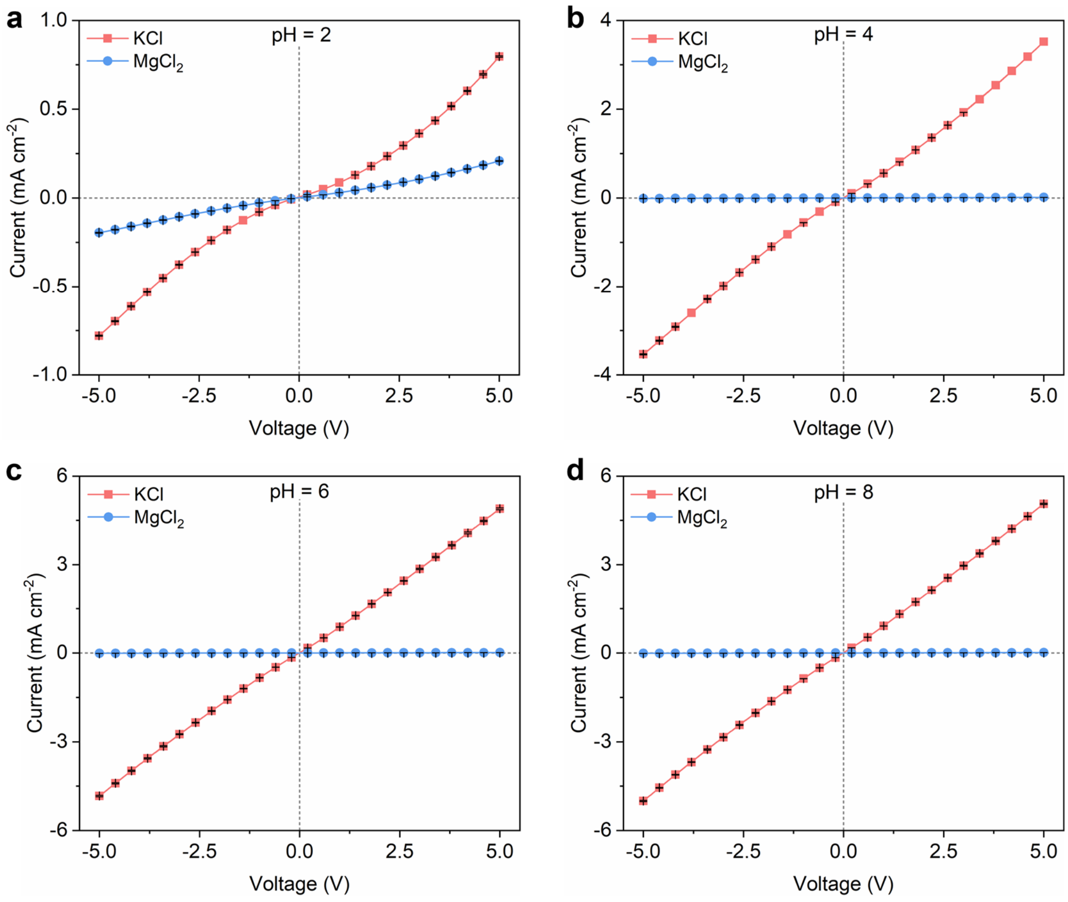

Supplementary Figure 19. Effect of pH on ion-selective transport of PEI-ICM. (a-d) *I*–*V* curves of PEI-ICM in 1 M KCl and 1 M $MgCl_2$ at pH values of 2.0 (a), 4.0 (b), 6.0 (c), and 8.0 (d), respectively.

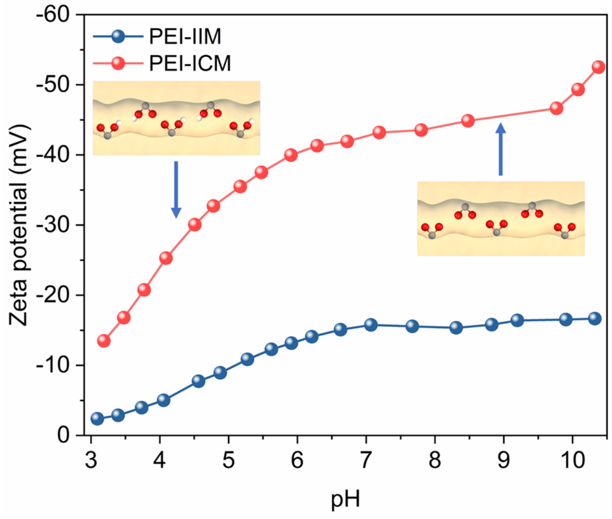

Supplementary Figure 20. Zeta potential of PEI-IIM and PEI-ICM as a function of pH. (Inset) At low pH, protonation of the surface carboxyl groups reduced the negative charge on the channel wall (left), while at high pH, deprotonation of these groups increased the negative charge on the channel wall (right). The substantially higher surface zeta potential of PEI-ICM compared to PEI-IIM suggests that UV-W process produces a significant amount of negatively charged groups (mainly carboxyl groups) on the membrane surface and within its channels.

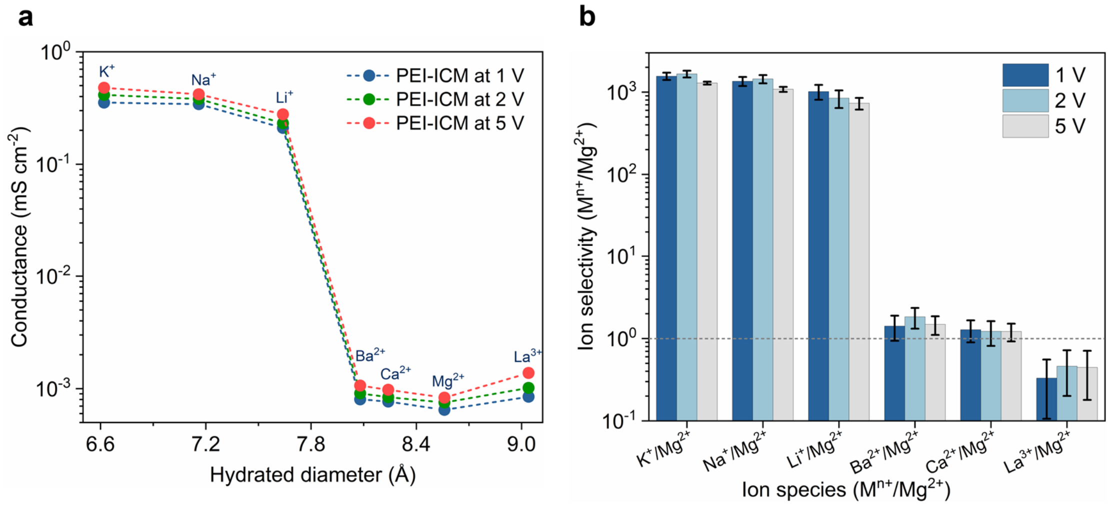

Supplementary Figure 21. Ion transport properties of PEI-ICM at different voltage. (a) The ion conductance of PEI-ICM at1 V, 2 V and 5 V. (b) The ion selectivity of the PEI-ICM at 1 V, 2 V and 5 V.

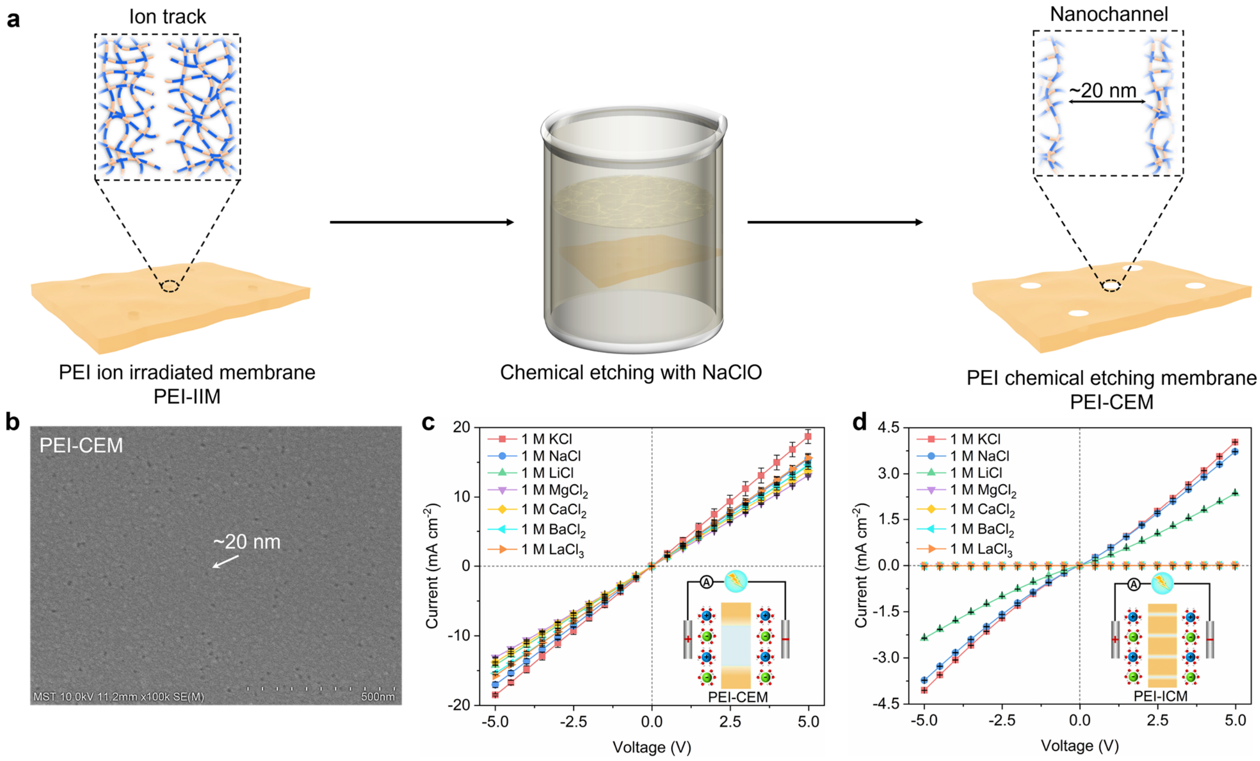

Supplementary Figure 22. Ion transport properties of PEI-ICM and PEI-CEM. (a) Schematic of PEI-CEM with channel size of 20 nm fabricated by chemical etching the PEI-IIM for 8 min with 5% NaClO solution at 50 °C under ultrasound treatment. (b) Surface SEM image of PEI-CEM. (c) *I–V* curves of PEI-CEM in various salt solutions. (d) *I–V* curves of PEI-ICM in corresponding salt solutions.

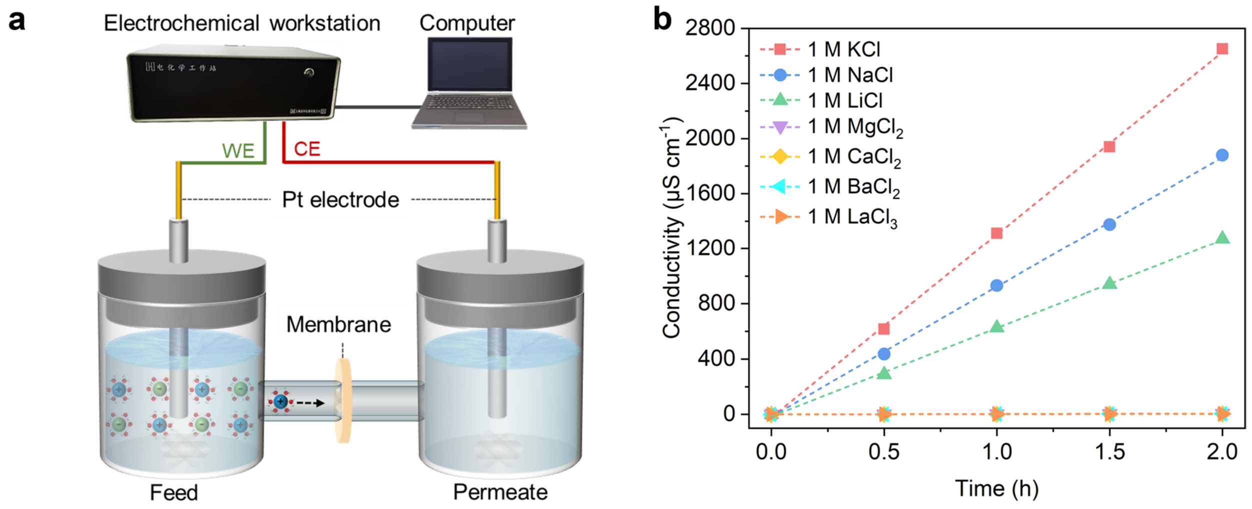

Supplementary Figure 23. Electrodialysis diffusion tests of PEI-ICM. (a) Schematic of experimental setup for electrodialysis ion permeation. (b) Permeate ion conductivity as a function of permeation time for PEI-ICM.

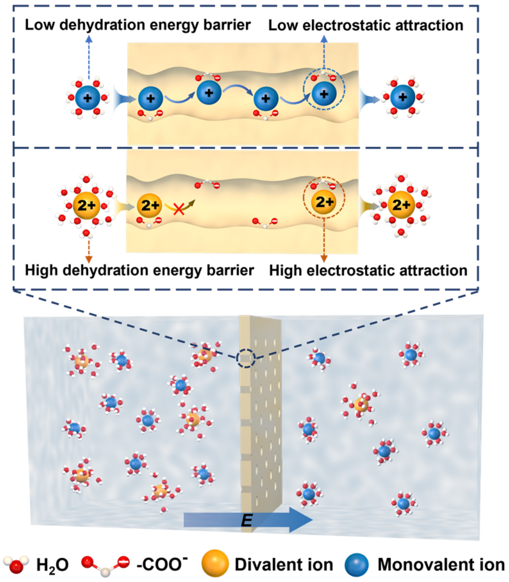

Supplementary Figure 24. Schematic of selective ion transport in channel stems from differences in the strengths of dehydration effect and Coulomb interaction between monovalent and divalent ions.

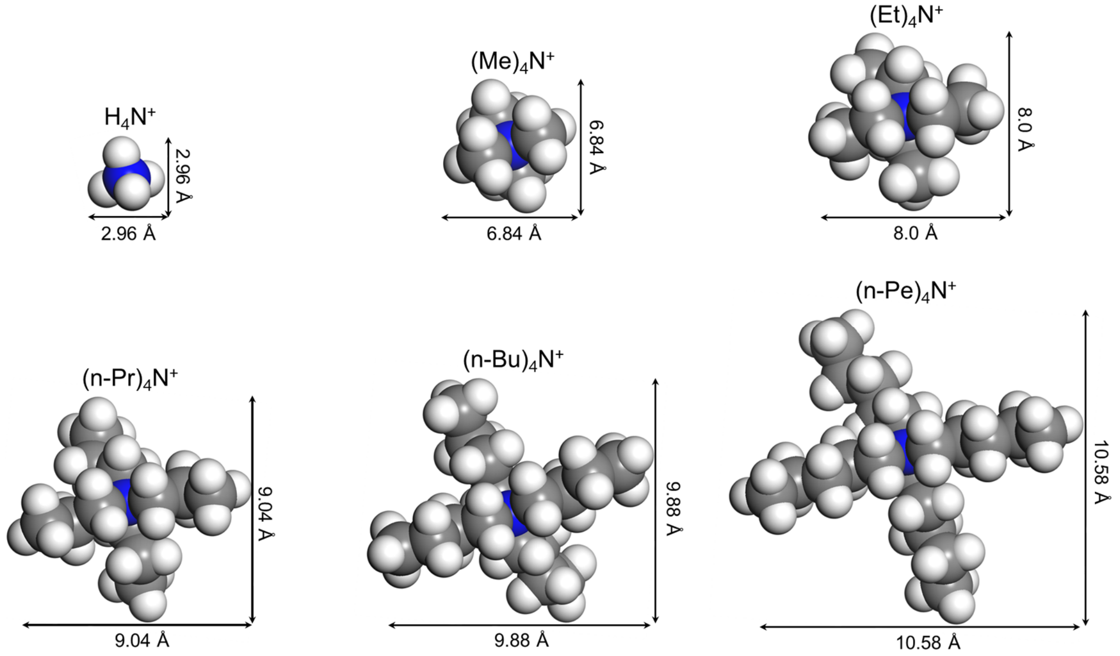

Supplementary Figure 25. Chemical structures and bare diameters of H_4N^+, $(Me)_4N^+$, $(Et)_4N^+$, $(n\text{-}Pr)_4N^+$, $(n\text{-}Bu)_4N^+$ and $(n\text{-}Pe)_4N^+$.

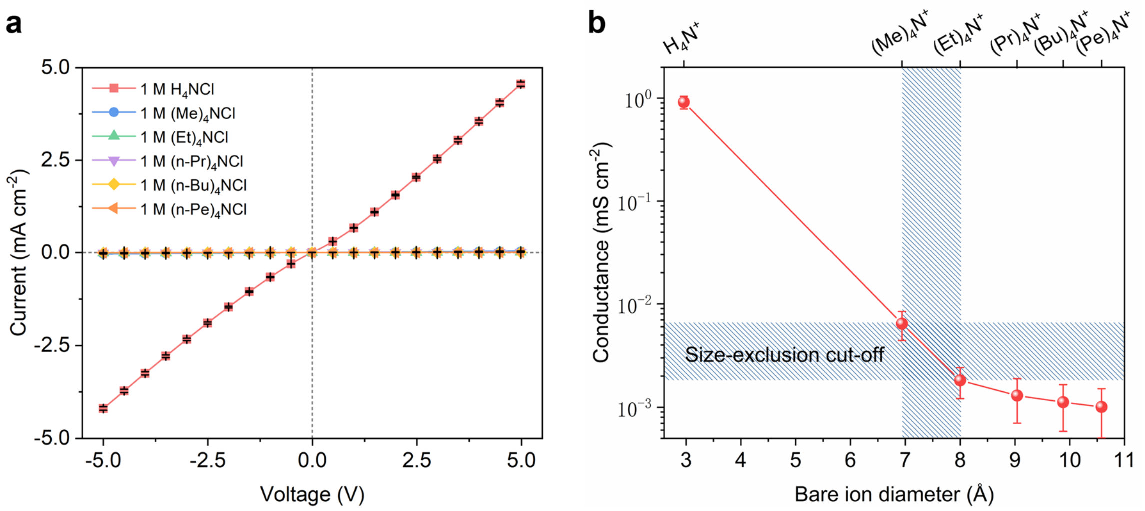

Supplementary Figure 26. Ion transport properties for PEI-ICM in quaternary ammonium chloride solutions. (a) *I*–*V* curves of PEI-ICM measured in a series of quaternary ammonium chloride solution. (b) Ion conductance of PEI-ICM for quaternary ammonium ions of varying sizes. For these quaternary ammonium ions with the identical charge, the ion conductance of PEI-ICM sharply decreased with the increase of ion diameter (particularly increased from 2.96 to 6.94 Å), revealing a pronounced size-exclusion cut-off behavior for the membrane channels.

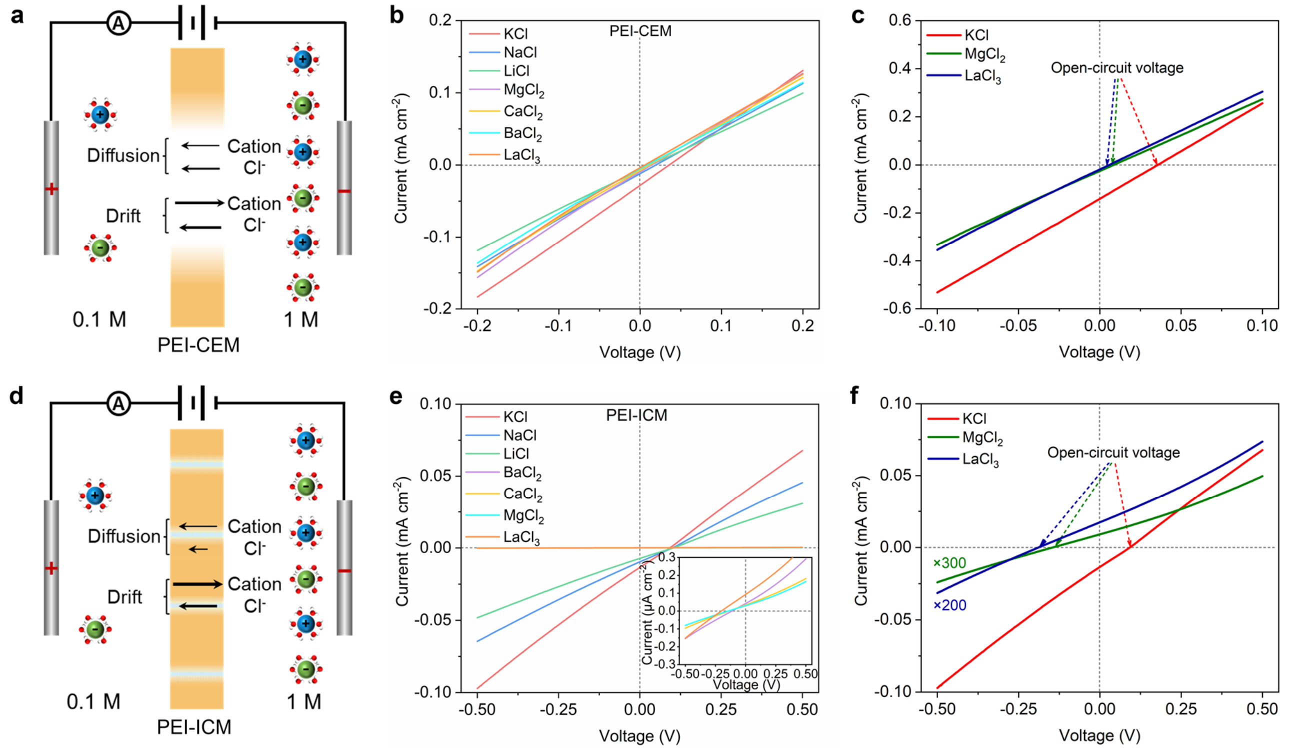

Supplementary Figure 27. Ion transport properties of PEI-CEM and PEI-ICM in asymmetric salt solutions. (a) Schematic of drift-diffusion measurements for PEI-CEM. (b) *I–V* curves of PEI-CEM measured in various chloride solutions with the concentration gradient of 10 across the membrane. (c) Examples of *I–V* curves for KCl, $MgCl_2$ and $LaCl_3$ show open-circuit voltages at 0.036V, 0.008 V and 0.006 V, respectively. (d) Schematic of drift-diffusion measurements for PEI-ICM. (e) *I–V* curves of PEI-ICM measured in various chloride solutions with the concentration gradient of 10 across the membrane. (Inset) Enlarged *I–V* curves measured in 1 M $BaCl_2$, $CaCl_2$, $MgCl_2$ and $LaCl_3$. (f) Examples of *I–V* curves for KCl, $MgCl_2$ and $LaCl_3$ show open-circuit voltages at 0.092V, -0.146 V and -0.19 V, respectively.

2.9 Ion separation performance of PEI-ICM (Figs. S28-S31)

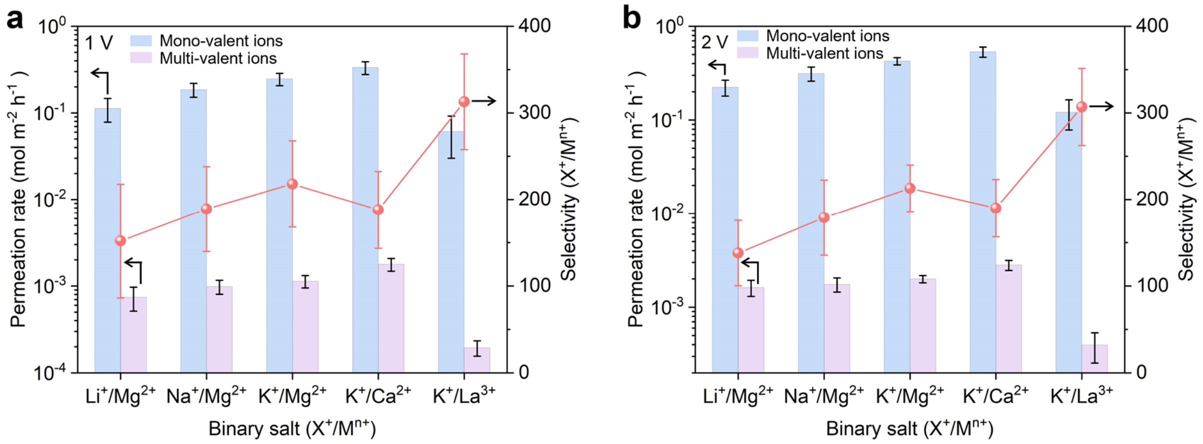

Supplementary Figure 28. Ion separation performance of PEI-ICM in binary salt solutions. (a, b) Ion permeation rate and selectivity performance of PEI-ICM under driving force of (a) 1 V and (b) 2 V.

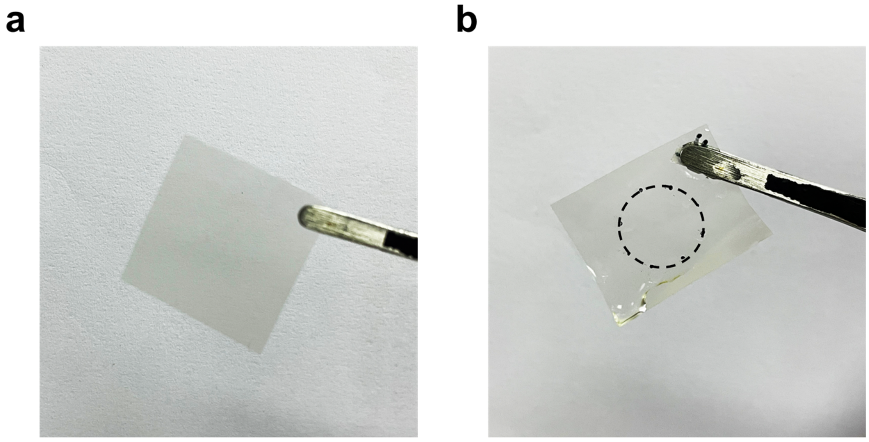

Supplementary Figure 29. (a), Digital photograph of PEI-ICM with 20 h UV-W process before long-term separation test. (b), Digital photograph of PEI-ICM with 20 h UV-W process after long-term separation test, the black dotted line is the actual membrane area used in the test.

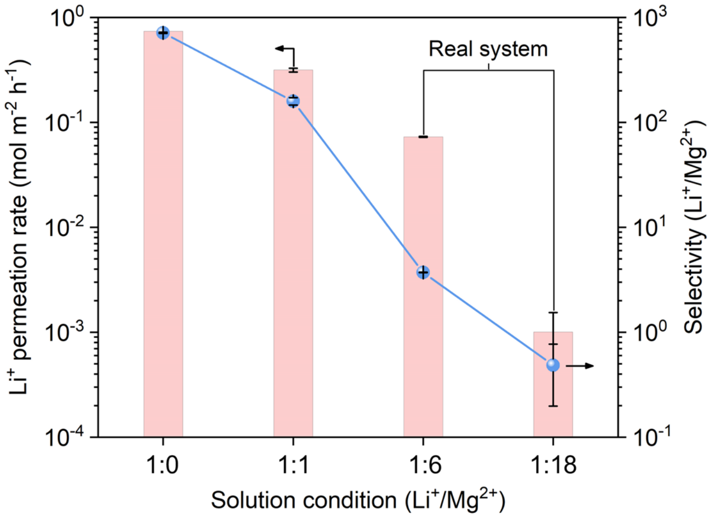

Supplementary Figure 30. Ion separation performance of PEI-ICM in various feed solution conditions (mol ratio of lithium to magnesium).

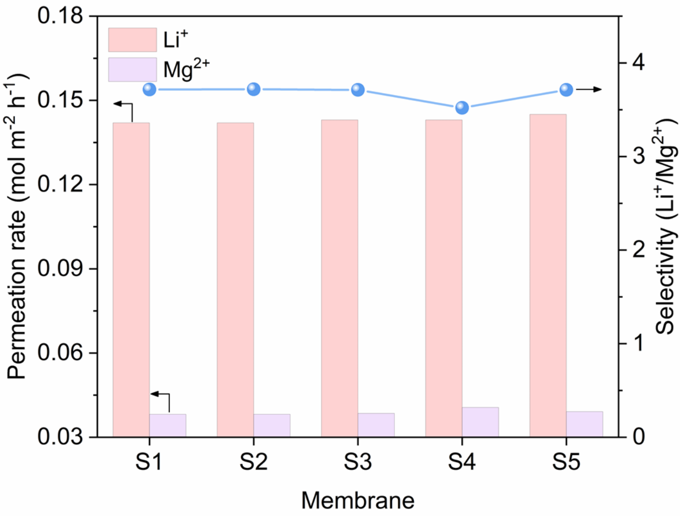

Supplementary Figure 31. Repeatability test of PEI-ICM. Li^+ permeation rate and Li^+/Mg^{2+} selectivity of various PEI-ICM samples in simulated brine water, under driving force of 1 V. The mass ratio of lithium to magnesium in the feed solution is 0.05 (LiCl 0.1wt%, $MgCl_2$ 2wt%).

3. Supplementary Tables

Supplementary Table 1.

The comparison of different swift heavy ions irradiation on PEI membranes.

	**^{40}Ar**	**^{86}Kr**	**^{181}Ta**
**Energy (GeV)**	2.0	2.15	2.444
**Energy loss (eV Å)**	49.7	288.3	1122
**Fluence rate (ions cm^{-2} s^{-1})**	––	~10^8	~10^7
**Projected range (μm)**	2360	505	202
**Track size (nm)**	––	~2 nm	~4 nm
**Damage core (nm)**	––	<1 nm	>1 nm
**Max fluence (ions cm^{-2})**	––	~10^{12}	~10^{11}
**Track structure**	Discontinuous	Narrow @continuous	Wide @continuous

Supplementary Table 2.

Detailed ion separation performance in single salt solutions for PEI-ICM.

**Feed solution**	**Concentration**	**Driving voltage**	**Permeation rate (mol m^{-2} h^{-1})**	**Selectivity (M^{n+}/Mg^{2+})**
KCl	1 M	5 V	3.01±0.086	1286.3
		2 V	1.132±0.0747	1659.8
		1 V	0.519±0.0374	1558.6
NaCl	1 M	5 V	2.53±0.127	1081.2
		2 V	0.984±0.08	1442.8
		1 V	0.451±0.04	1354.4
LiCl	1 M	5 V	1.71±0.195	730.8
		2 V	0.576±0.098	844.6
		1 V	0.301±0.049	1024
$BaCl_2$	1 M	5 V	0.00347±0.00063	1.483
		2 V	0.00125±0.00025	1.833
		1 V	0.00047±0.00011	1.417
$CaCl_2$	1 M	5 V	0.00285±0.00049	1.218
		2 V	0.00083±0.0002	1.22
		1 V	0.00043±0.00009	1.279
$MgCl_2$	1 M	5 V	0.00234±0.00057	––
		2 V	0.00068±0.00014	––
		1 V	0.00033±0.00007	––
$LaCl_3$	1 M	5 V	0.00104±0.00044	0.444
		2 V	0.00031±0.00013	0.460
		1 V	0.00011±0.00005	0.330

Supplementary Table 3.

Experimental ions and their hydrated diameters and hydration energies [6, 7].

Ions	Bare diameter (Å)	Hydrated diameter (Å)	Hydration energy ($-kJ\ mol^{-1}$)
H_3O^+	2.3	5.64	--
K^+	2.66	6.62	295
Na^+	1.90	7.16	365
Li^+	1.20	7.64	475
Ba^{2+}	2.846	8.08	1250
Ca^{2+}	1.98	8.24	1505
Mg^{2+}	1.30	8.56	1830
La^{3+}	2.3	9.04	3145
Cl^-	3.62	6.64	340
OH^-	3.52	6.00	430
H_4N^+	2.96	6.62	285
$(Me)_4N^+$	6.94	7.34	160
$(Et)_4N^+$	8.00	8.00	0
$(n\text{-}Pr)_4N^+$	9.04	9.04	0
$(n\text{-}Bu)_4N^+$	9.88	9.88	0
$(n\text{-}Pe)_4N^+$	10.58	10.58	0

Supplementary Table 4.

Detailed ion separation performance for PEI-ICM in binary salt solutions.

Feed solution	Concentration	Driving voltage	Permeation rate (mol m^{-2} h^{-1})	Selectivity
LiCl & $MgCl_2$	0.5 M + 0.5 M	5 V	0.379±0.063	147
		2 V	0.223±0.043	138
		1 V	0.112±0.034	152
NaCl & $MgCl_2$	0.5 M + 0.5 M	5 V	0.47±0.083	182
		2 V	0.31±0.054	179
		1 V	0.186±0.034	189
KCl & $MgCl_2$	0.5 M + 0.5 M	5 V	0.552±0.080	204
		2 V	0.425±0.038	213
		1 V	0.247±0.04	218
KCl & $CaCl_2$	0.5 M + 0.5 M	5 V	0.67±0.073	187
		2 V	0.533±0.066	190
		1 V	0.334±0.056	188
KCl & $LaCl_3$	0.5 M + 0.5 M	5 V	0.26±0.055	290
		2 V	0.121±0.043	307
		1 V	0.06±0.031	313

Supplementary Table 5.

The comparison of Li^+/Mg^{2+} actual separation performance for separation membrane.

Membranes	Feed solution ($LiCl + MgCl_2$)	Li^+ permeation rate ($mol\ m^{-2}\ h^{-1}$)	Selectivity (Li^+/Mg^{2+})	Ref
TFC	0.1 M + 0.1 M	1.6	5.3	[8]
TFN-(Zr)-1	0.1 M + 0.1 M	1.7	6.8	[8]
TFN-(Zr/Ti)-1	0.1 M + 0.1 M	1.7	8.2	[8]
TFN-(Zr)-2	0.1 M + 0.1 M	1.9	12	[8]
TFN-(Zr/Ti)-2	0.1 M + 0.1 M	2.0	11	[8]
UiO-66-NH_2 LLM-1	0.1 M + 0.1 M	0.045	25	[9]
UiO-66-NH_2 LLM-2	0.1 M + 0.1 M	0.045	39	[9]
UiO-66-NH_2 LLM-3	0.1 M + 0.1 M	0.069	39	[9]
UiO-66-NH_2 LLM-4	0.1 M + 0.1 M	0.072	65	[9]
M-0.6	0.1 M + 0.1 M	0.328	8.99	[10]
UiO-66	0.1 M + 0.1 M	0.074	19	[11]
UiO-66-SO_3H (10%)	0.1 M + 0.1 M	0.078	4	[11]
UiO-66-SO_3H (25%)	0.1 M + 0.1 M	0.10	1.9	[11]
UiO-67	0.5 M + 0.5 M	7.22	81	[12]
COF-EB_1BD_1/PAN	0.1 M + 0.1 M	0.045	443	[13]
$TpBDMe_2$	0.1 M + 0.1 M	0.0383	35.8	[14]
COF-4EO-PAN	1 M + 1 M	0.23	56	[15]
CC3 membrane	0.1 M + 0.1 M	0.077	100	[16]
QPO/DAN-SA-5	0.1 M + 0.1 M	0.168	16.5	[17]
Neosepta^{TM} CIMS	0.1 M + 0.1 M	0.0299	3.5	[17]
ENF-Q3	0.1 M + 0.1 M	2.08	11.3	[18]

CSO	0.1 M + 0.1 M	2.23	1.6	[18]
SPES-composited	0.25 M + 0.25 M	0.12	5	[19]
P-COOH	0.1 M + 0.1 M	0.4	1.5	[20]
P-COOH-Q	0.1 M + 0.1 M	0.19	3.8	[20]
P-COOH-QS	0.1 M + 0.1 M	0.40	1.7	[20]
P-COOH-QSQ	0.1 M + 0.1 M	0.16	5.16	[20]
SPPO	0.1 M + 0.1 M	0.43	0.68	[21]
SPQ-10	0.1 M + 0.1 M	0.89	12.7	[21]
PET Lumirror$^{@}$	0.5 M + 0.5 M	0.0143	21.21	[22]
QAIPA-10	0.1 M + 0.1 M	0.006	5.2	[23]
QAIPA -15	0.1 M + 0.1 M	0.013	20	[23]
QAIPA -20	0.1 M + 0.1 M	0.12	8	[23]
PIM-DB18C6-TB	0.1 M + 0.1 M	0.0812	24.09	[24]
PEI	0.1 M + 0.1 M	0.00086	14	[25]
rGO	0.1 M + 0.1 M	0.015	12	[26]
MXene @PSS	0.2 M + 0.2 M	0.08	25	[27]
2D SEP-MMTM	0.1 M + 0.1 M	0.28	7.3	[28]
GO-PEI	0.5 M + 0.5 M	0.067	16.8	[29]
	0.5 M + 0.5 M	0.380 (5 V)	147	
PEI-ICM	0.5 M + 0.5 M	0.223 (2 V)	138	This work
	0.5 M + 0.5 M	0.112 (1 V)	152	

Supplementary Table 6.

Detailed lithium extraction performance for PEI-ICM in simulated brine water.

**Feed solution**	**Feed $R_{M/L}$ (ω_{Mg}/ω_{Li})**	**Driving voltage**	**Li^+ permeation rate ($mol\ m^{-2}\ h^{-1}$)**	**Separation factor**
Li^+: 0.1wt% Mg^{2+}: 2wt%	20	1 V	0.8867	10.64
Li^+: 0.025wt% Mg^{2+}: 1.5wt%	60	1 V	0.006142	10.34

Supplementary Table 7.

The comparison of Li^{+} extraction performance from simulated brine water for polymer membrane.

Membranes	Membrane process	Operation stage	Feed $R_{M/L}$	Separation factor	Ref
**Desal 5 DL**	NF	1	20.2	3.5	[30]
**Desal DK**	NF	1	24	3.2	[31]
**Desal DL-2540**	NF	1	64	3.3	[32]
**BAPP/TMC/PAN**	NF	1	20	2.6	[33]
**PEI/TMC/PES**	NF	1	20	15	[34]
**PES/CNC-COOH/PA**	NF	1	30	12.15	[35]
**PES/CNC-COOH/PA**	NF	1	60	5.84	[35]
**MPMC**	NF	1	21.4	7.1	[36]
**(PES-GO)/PEI/TMC**	NF	1	20	16.1	[37]
**PA-B2-E3**	NF	1	24	9.2	[38]
**XN45 in EALNF**	NF	1	20	830.98	[39]
**Selemion CSO**	ED	1	20.7	10	[40]
**CIMS, ACS**	ED	10	60	8.57	[41]
**CSO, ASA**	ED	10	9.85	17.3	[42]
**CIMS, ACS**	ED	10	16.07	2.86	[43]
**CIMS, ACS**	ED	10	35.18	9	[43]
**CIMS, ACS**	ED	10	54.3	5.95	[43]
**CIMS, ACS**	ED	10	72.72	4.3	[43]
**PEI-ICM**	ED	1	20	10.64	This work
**PEI-ICM**	ED	1	60	10.34	This work

4. References

1. Schiwietz G, Czerski K, Roth M, et al. Femtosecond dynamics–snapshots of the early ion-track evolution. *Nuclear Instruments and Methods in Physics Research Section B: Beam Interactions with Materials and Atoms*. 2004; **225(1-2):** 4-26.
2. Polvi J, Nordlund K. Low-energy irradiation effects in cellulose. *Journal of Applied Physics*. 2014; **115**(2).
3. Korolkov IV, Mashentseva AA, Güven O *et al.* The effect of oxidizing agents/systems on the properties of track-etched PET membranes. *Polymer Degradation and Stability*. 2014; **107**: 150-157.
4. Sabrina Carroccio CP. Comparison of Photooxidation and Thermal Oxidation Processes in Poly(ether imide). *Macromolecules*. 2005; **38**: 6863-6870.
5. Li Y, Liu Y, Liu S *et al.* Photoaging of Baby Bottle-Derived Polyethersulfone and Polyphenylsulfone Microplastics and the Resulting Bisphenol S Release. *Environ Sci Technol*. 2022; **56**(5): 3033-3044.
6. Marcus Y. A simple empirical model describing the thermodynamics of hydration of ions of widely varying charges, sizes, and shapes. *Biophysical chemistry*. 1994; **51**(2-3): 111-127.
7. Nightingale Jr E. Phenomenological theory of ion solvation. Effective radii of hydrated ions. *The Journal of Physical Chemistry*. 1959; **63**(9): 1381-1387.
8. Xu T, Sheng F, Wu B, et al. Ti-exchanged UiO-66-NH_2-containing polyamide membranes with remarkable cation permselectivity. *Journal of Membrane Science*. 2020; **615**: 118608.
9. Xu T, Shehzad M A, Yu D, et al. Highly cation permselective metal-organic framework membranes with leaf-like morphology. *Chemsuschem*. 2019; **12(12)**: 2593-2597.
10. Tao L, Wang X, Wu F, et al. Highly efficient Li^+/Mg^{2+} separation of monovalent cation permselective membrane enhanced by 2D metal organic framework nanosheets. *Separation and Purification Technology*. 2022; **296:** 121309.

11. Xu, T.; Shehzad, M. A.; Wang, X.; Wu, B.; Ge, L.; Xu, T., Engineering leaf-like UiO-66-SO_3H membranes for selective transport of cations. *Nano-micro Lett.* 2020; **12(1):** 1-11.
12. Xu R, Kang Y, Zhang W, et al. Oriented UiO-67 metal–organic framework membrane with fast and selective lithium-ion transport. *Angewandte Chemie*. 2022; **61(3):** e202115443.
13. Hou L, Xian W, Bing S, et al. Understanding the ion transport behavior across nanofluidic membranes in response to the charge variations. *Advanced Functional Materials*. 2021; **31(16):** 2009970.
14. Sheng F, Wu B, Li X, et al. Efficient ion sieving in covalent organic framework membranes with sub-2-nanometer channels. *Advanced Materials*. 2021; **33(44):** 2104404.
15. Bing S, Xian W, Chen S, et al. Bio-inspired construction of ion conductive pathway in covalent organic framework membranes for efficient lithium extraction. *Matter*. 2021; **4(6):** 2027-2038.
16. Xu T, Wu B, Hou L, et al. Highly ion-permselective porous organic cage membranes with hierarchical channels. *Journal of the American Chemical Society*. 2022; **144(23):** 10220-10229.
17. Irfan M, Xu T, Ge L *et al.* Zwitterion structure membrane provides high monovalent/divalent cation electrodialysis selectivity: Investigating the effect of functional groups and operating parameters. *Journal of Membrane Science*. 2019; **588**: 117211.
18. Sheng F, Hou L, Wang X *et al.* Electro-nanofiltration membranes with positively charged polyamide layer for cations separation. *Journal of Membrane Science*. 2020; **594**: 117453.
19. Saif H, Huertas R, Pawlowski S *et al.* Development of highly selective composite polymeric membranes for Li^+/Mg^{2+} separation. *Journal of Membrane Science*. 2021; **620**: 118891.
20. Afsar NU, Shehzad MA, Irfan M *et al.* Cation exchange membrane integrated with cationic and anionic layers for selective ion separation via electrodialysis. *Desalination*. 2019; **458**: 25-33.
21. Afsar NU, Ji W, Wu B *et al.* SPPO-based cation exchange membranes with a positively charged layer for cation fractionation. *Desalination*. 2019; **472**: 114145.

22. Wang P, Wang M, Liu F *et al.* Ultrafast ion sieving using nanoporous polymeric membranes. *Nature communications*. 2018; **9**(1): 569.
23. Afsar NU, Ge X, Zhao Z *et al.* Zwitterion membranes for selective cation separation via electrodialysis. *Separation and Purification Technology*. 2021; **254**: 117619.
24. Dong Y, Liu Y, Li H *et al.* Crown ether-based Tröger's base membranes for efficient Li^+/Mg^{2+} separation. *Journal of Membrane Science*. 2023; **665**: 121113.
25. Guo Z, Li F, Wu X *et al.* Efficient ion sieving and ion transport properties in sub-nanoporous polyetherimide membranes. *Desalination*. 2024; **573:** 117192.
26. Xi Y H, Liu Z, Ji J, et al. Graphene-based membranes with uniform 2D nanochannels for precise sieving of mono-/multi-valent metal ions. *Journal of Membrane Science*. 2018; **550:** 208-218.
27. Lu Z, Wu Y, Ding L, et al. A lamellar MXene ($Ti_3C_2T_x$)/PSS composite membrane for fast and selective lithium-ion separation. *Angewandte Chemie*. 2021; **133(41):** 22439-22443.
28. Wen T, Zhao Y, Wang X, et al. Efficient and ultrafast separation of Li^+ and Mg^{2+} by the porous two-dimensional nanochannel of perm-selective montmorillonite membrane. *Chemical Engineering Journal*. 2023; **475:** 146101.
29. Zhang W, Huang Q, Liu S, et al. Graphene oxide membrane regulated by surface charges and interlayer channels for selective transport of monovalent ions over divalent ions. *Separation and Purification Technology*. 2022; **291:** 120938.
30. Wen X, Ma P, Zhu C *et al.* Preliminary study on recovering lithium chloride from lithium-containing waters by nanofiltration. *Separation and purification technology*. 2006; **49**(3): 230-236.
31. Gang Y, Hong S, Wenqiang L *et al.* Investigation of Mg^{2+}/Li^+ separation by nanofiltration. *Chinese Journal of Chemical Engineering*. 2011; **19**(4): 586-591.
32. Sun S, Cai L, Nie X *et al.* Separation of magnesium and lithium from brine using a Desal nanofiltration membrane. *Journal of Water Process Engineering*. 2015; **7**: 210-217.

33. Li X, Zhang C, Zhang S *et al.* Preparation and characterization of positively charged polyamide composite nanofiltration hollow fiber membrane for lithium and magnesium separation. *Desalination*. 2015; **369**: 26-36.

34. Xu P, Wang W, Qian X *et al.* Positive charged PEI-TMC composite nanofiltration membrane for separation of Li^+ and Mg^{2+} from brine with high Mg^{2+}/Li^+ ratio. *Desalination*. 2019; **449**: 57-68.

35. Guo C, Li N, Qian X *et al.* Ultra-thin double Janus nanofiltration membrane for separation of Li^+ and Mg^{2+}: "Drag" effect from carboxyl-containing negative interlayer. *Separation and Purification Technology*. 2020; **230**: 115567.

36. Zhang H, Xu Z, Ding H *et al.* Positively charged capillary nanofiltration membrane with high rejection for Mg^{2+} and Ca^{2+} and good separation for Mg^{2+} and Li^+. *Desalination*. 2017; **420**: 158-166.

37. Xu P, Hong J, Qian X *et al.* "Bridge" graphene oxide modified positive charged nanofiltration thin membrane with high efficiency for Mg^{2+}/Li^+ separation. *Desalination*. 2020; **488**: 114522.

38. Li W, Shi C, Zhou A *et al.* A positively charged composite nanofiltration membrane modified by EDTA for $LiCl/MgCl_2$ separation. *Separation and Purification Technology*. 2017; **186**: 233-242.

39. Yong M, Tang M, Sun L *et al.* Sustainable lithium extraction and magnesium hydroxide co-production from salt-lake brines. *Nature Sustainability*. 2024; **7(12):** 1662-1671.

40. Nie X, Sun S, Song X *et al.* Further investigation into lithium recovery from salt lake brines with different feed characteristics by electrodialysis. *Journal of Membrane Science*. 2017; **530**: 185-191.

41. Ji Z, Chen Q, Yuan J *et al.* Preliminary study on recovering lithium from high Mg^{2+}/Li^+ ratio brines by electrodialysis. *Separation and Purification Technology*. 2017; **172**: 168-177.

42. Ying J, Luo M, Jin Y *et al.* Selective separation of lithium from high Mg/Li ratio brine using single-stage and multi-stage selective electrodialysis processes. *Desalination*. 2020; **492**: 114621.

43. Guo Z, Ji Z, Chen Q *et al.* Prefractionation of LiCl from concentrated seawater/salt lake brines by electrodialysis with monovalent selective ion exchange membranes. *Journal of Cleaner Production*. 2018; **193**: 338-350.
